# A severe atherosclerosis mouse model on the resistant NOD background

**DOI:** 10.1242/dmm.033852

**Published:** 2018-10-08

**Authors:** Xugang Wang, Rong Huang, Lichen Zhang, Saichao Li, Jing Luo, Yanrong Gu, Zhijun Chen, Qianqian Zheng, Tianzhu Chao, Wenping Zheng, Xinhui Qi, Li Wang, Yinhang Wen, Yinming Liang, Liaoxun Lu

**Affiliations:** 1 Laboratory of Genetic Regulators in the Immune System, Henan Collaborative Innovation Center of Molecular Diagnosis and Laboratory Medicine, School of Laboratory Medicine, Xinxiang Medical University, Henan Province 453003, China; 2 Henan Key Laboratory of Immunology and Targeted Therapy, School of Laboratory Medicine, Xinxiang Medical University, Henan Province 453003, China; 3 Laboratory of Mouse Genetics, Institute of Psychiatry and Neuroscience, Xinxiang Medical University, Henan Province 453003, China

**Keywords:** NOD, Atherosclerosis, CRISPR/Cas9, ApoE, LDLR

## Abstract

Atherosclerosis is a complex disease affecting arterial blood vessels and blood flow that could result in a variety of life-threatening consequences. Disease models with diverged genomes are necessary for understanding the genetic architecture of this complex disease. Non-obese diabetic (NOD) mice are highly polymorphic and widely used for studies of type 1 diabetes and autoimmunity. Understanding atherosclerosis development in the NOD strain is of particular interest as human atherosclerosis on the diabetic and autoimmune background has not been successfully modeled. In this study, we used CRISPR/Cas9 genome editing to genetically disrupt apolipoprotein E (ApoE) and low-density lipoprotein receptor (LDLR) expression on the pure NOD background, and compared phenotype between single-gene-deleted mice and double-knockout mutants with reference to ApoE-deficient C57BL/6 mice. We found that genetic ablation of *Ldlr* or *Apoe* in NOD mice was not sufficient to establish an atherosclerosis model, in contrast to ApoE-deficient C57BL/6 mice fed a high-fat diet (HFD) for over 12 weeks. We further obtained NOD mice deficient in both LDLR and ApoE, and assessed the severity of atherosclerosis and immune response to hyperlipidemia in comparison to ApoE-deficient C57BL/6 mice. Strikingly, the double-knockout NOD mice treated with a HFD developed severe atherosclerosis with aorta narrowed by over 60% by plaques, accompanied by destruction of pancreatic islets and an inflammatory response to hyperlipidemia. Therefore, we succeeded in obtaining a genetic model with severe atherosclerosis on the NOD background, which is highly resistant to the disease. This model is useful for the study of atherosclerosis in the setting of autoimmunity.

## Abstract (precis)

**Summary:** Double knockout of *Apoe* and *Ldlr* on the highly atherosclerosis-resistant NOD mouse background results in severe atherosclerosis, which paves the way for the study of severe atherosclerosis in the setting of autoimmunity.

## INTRODUCTION

Atherosclerosis results from chronic inflammation and remains the top-ranking cause of human mortality (Lusis, 2000). Detailed mechanism studies of atherosclerosis could be facilitated by animal models; however, because atherosclerosis is a complex disease influenced by various genetic factors and their interaction with environmental factors (Srivastava et al., 2012), more diverged genome backgrounds are required for its study. In humans, atherosclerosis can develop in combination with an autoimmune disease, which itself could be an exacerbating factor of atherosclerosis. Mouse strains harboring natural variations have contributed to uncovering such genetic factors, but atherosclerosis in mice with spontaneous autoimmunity has not been successfully modeled (Smith, 2003; Lusis, 2012; Bennett et al., 2015; Grainger et al., 2016). Non-obese diabetic (NOD) mice, specifically the NOD/ShiLtJ strain, which spontaneously develops type 1 diabetes (T1D) and autoimmunity affecting multiple organs, including the pancreas and thyroid, provide a desirable setting for the study of human atherosclerosis with T1D and autoimmunity (Anderson and Bluestone, 2005; Pearson et al., 2016). From a genetics perspective, it is tempting to first characterize the atherosclerosis development on the NOD background, which possesses over 4-million single nucleotide polymorphisms (SNPs) (Keane et al., 2011). It is important to note that backcrossing of the *Apoe*-deficient allele to the NOD background could result in residual genomic components, named passenger DNA, from the donor strain (e.g. C57BL/6), which could modify the phenotype (Ridgway, 2014). The CRISPR/Cas9 genome-editing tool allows for genetic manipulation of the fertilized oocytes of NOD mice without involvement of backcrossing and completely avoids the problem of passenger-DNA-mediated phenotype ambiguity. Therefore, it is optimal to disrupt genes essential for lipid metabolism on the pure NOD background and validate the development of atherosclerosis. A second interesting point concerning the NOD background is that this strain spontaneously develops T1D originating from pathological T-cell reactivity to pancreatic tissue, as well as other autoimmune diseases. In humans, T1D occurrence was found to be associated with atherosclerosis; however, the predominant T1D model, NOD mice, have not been genetically engineered to model pancreatic dysfunction accompanied by atherosclerosis. Efforts have been made to develop atherosclerotic mice with diabetes, but these have failed with NOD mice probably due to the resistance of this strain to drug-induced atherosclerosis (Keren et al., 2001). There is currently a lack of genetic models of atherosclerosis in the setting of autoimmunity, mainly because it is difficult to develop atherosclerosis in NOD wild-type mice. It is necessary to perform genetic disruption of gene(s) by genome editing on the pure NOD background. In this study, to genetically validate development of atherosclerosis on the pure NOD background we used the CRISPR/Cas9 genome-editing tool to target *Apoe* and *Ldlr* loci. Our experiments showed that NOD mice are highly resistant to high-fat diet (HFD)-induced atherosclerosis regardless of genetic ablation of *Apoe* or *Ldlr*. However, strikingly, the double-knockout mice deficient in both ApoE and LDLR developed severe HFD-induced atherosclerosis. In these novel models on a pure NOD background, we found that single-gene deficiency of *Apoe* or *Ldlr* had significantly lower serum lipids than *Apoe*-deficient C57BL/6 mice; however, the double-knockout animals on the NOD background had higher lipid levels. The double-knockout mice had a pro-inflammatory immune response to hyperlipidemia, and severe destruction of pancreatic islets. Such a model developed on the resistant NOD background could be particularly valuable to study atherosclerosis with complication of autoimmunity such as T1D (Eckel and Eisenbarth, 2012; Mitchell, 2012).

## RESULTS

### Genetic ablation of ApoE and LDLR in NOD mice by the CRISPR/Cas9 system

**Fig. 1.**
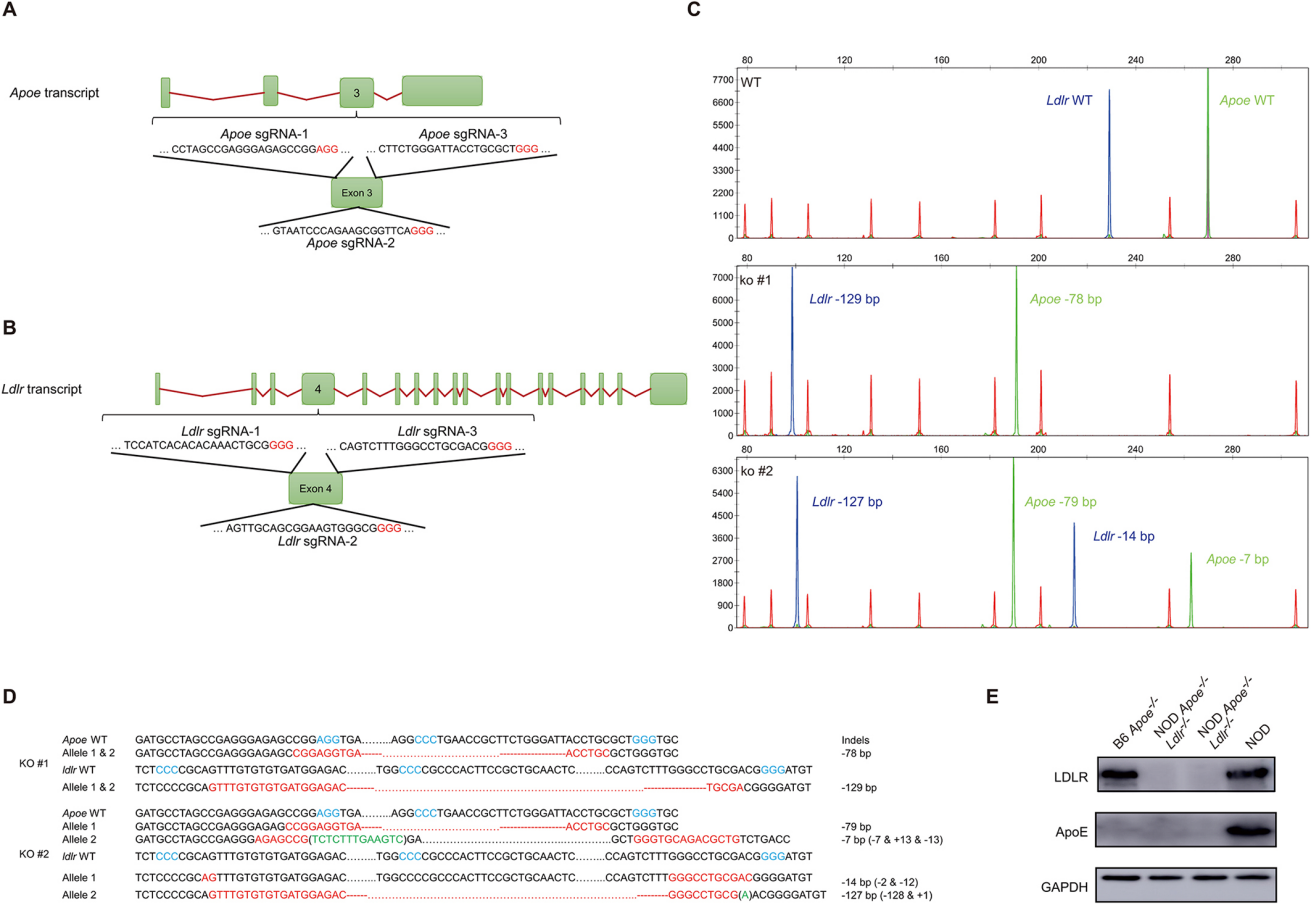
**Genetic ablation of ApoE and LDLR in** **the** **NOD mouse by** **the** **CRISPR/Cas9 system.** (A,B) Targeting design and schematic presentation of intron and exon structure for *Apoe* and *Ldlr*. Three sgRNAs were selected for each gene and protospacer adjacent motifs (PAMs) are shown in red letters. (C) Genotyping of F0 mouse by fluorescent PCR and capillary electrophoresis analysis (CEA). Mouse tail genomic DNA samples were isolated and subjected to fluorescent PCR amplification followed by CEA detection. The size markers were labeled by rhodamine-X (ROX; red), amplicons of the *Apoe* locus were HEX (green) labeled, and *Ldlr* targeted locus was amplified with FAM (blue)-labeled primers. (D) Sanger sequencing validation of the mutant mice. The PAM motifs are shown in blue letters, deletions in red letters and insertions in green letters. (E) Western blot of ApoE and LDLR protein expression in mutant mice.

Previous studies suggested that NOD mice could be resistant to HFD-induced atherosclerosis; however, genetic validation of atherosclerosis on the pure NOD background deficient in ApoE or LDLR was not performed (Keren et al., 2001). In our study, the NOD ApoE- or LDLR-deficient mice were generated by targeting the *Apoe* and *Ldlr* genes in NOD fertilized eggs by using the CRISPR/Cas9 system (Fig. 1A,B), as described in the literature and in the Materials and Methods section (Yang et al., 2014). We started with the aim of obtaining double-knockout animals, and co-injected the single-guide RNAs (sgRNAs) together with the other CRISPR/Cas9 effector components into fertilized eggs collected from super-ovulated NOD mice in procedures illustrated in Fig. S1A. To screen for mutant mice in the F0 newborns, which could harbor mutant alleles for both *Apoe* and *Ldlr* genes, we designed duplex detection for fluorescent PCRs amplifying the targeted loci. The two isolated founder males with bi-allelic mutation for both *Apoe* and *Ldlr* genes were crossed to NOD wild-type females followed by intercrosses to obtain F2 homozygotes. As shown in Fig. 1C, the wild-type *Apoe* allele was 268 bp (indicated in green) and the wild-type *Ldlr* allele was 230 bp (indicated in blue), and we found that one representative F0 animal had a bi-allelic 78 bp deletion at the *Apoe* locus, and bi-allelic 129 bp deletion at the *Ldlr* locus, while another representative animal had deletions of 7 bp/79 bp at the *Apoe* locus and 14 bp/127 bp at the *Ldlr* locus. The deletions were confirmed by Sanger sequencing (Fig. 1D and Fig. S1B,C). The F0 founder mice carrying loss of open reading frame (ORF) mutant alleles were backcrossed to NOD wild-type mice and the resultant F1 mice were intercrossed to produce F2 animals in which *Apoe* and *Ldlr* loci could be homozygous mutant for the two genes or be segregated for single-gene knockout used in further experiments. Backcrosses and intercrosses were performed to minimize potential off-target mutations, and we compared F2 animals homozygous for both the *Apoe* and *Ldlr* mutation to their wild-type counterparts to segregate unexpected mutations (Harel et al., 2015). The western blot experiments validated the protein expression of the selected mutants (Fig. 1E).

### NOD mice are highly resistant to atherosclerosis in the absence of ApoE or LDLR

**Fig. 2.**
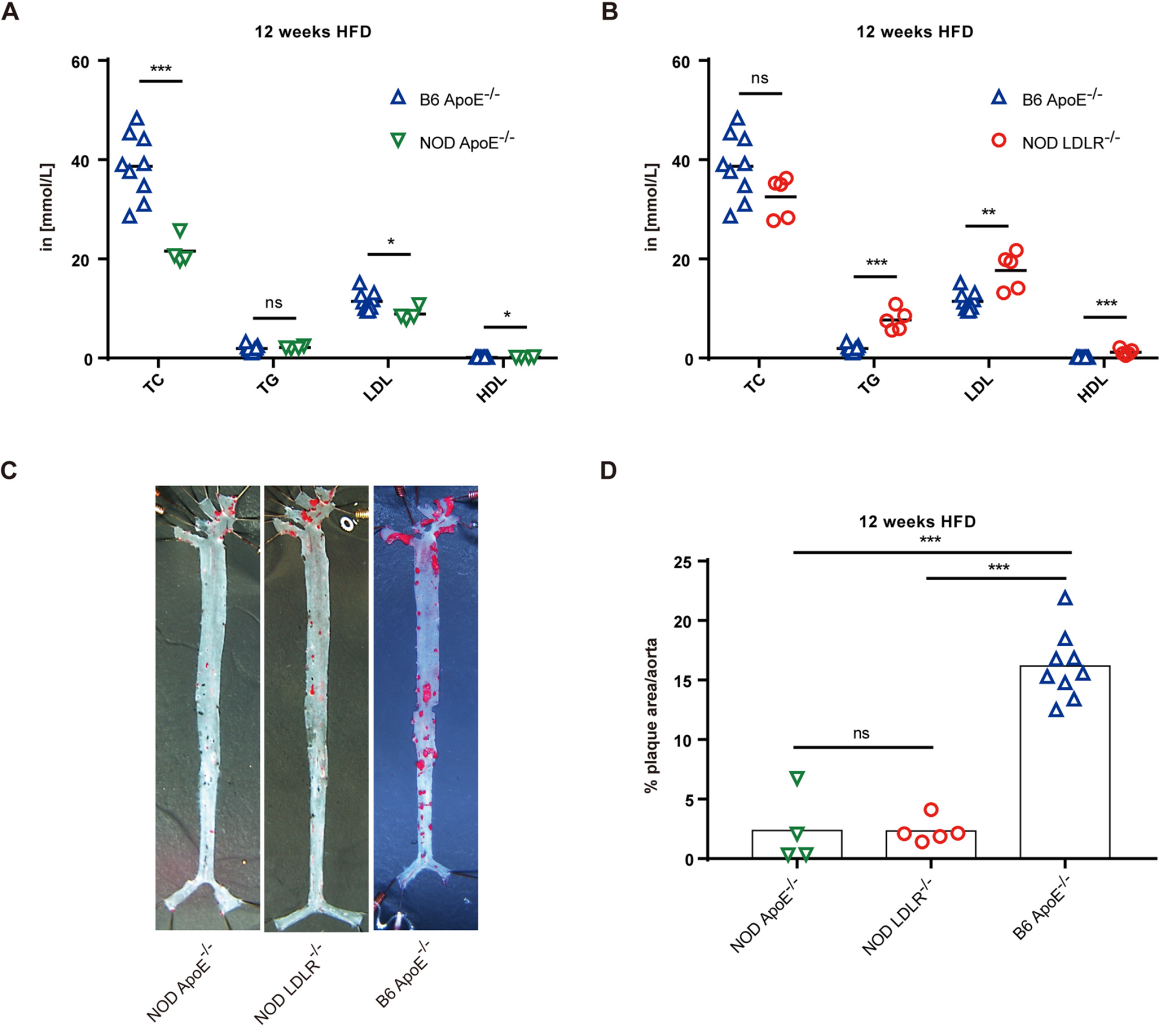
**Atherosclerosis development in ApoE- or LDLR-deficient NOD mice.** (A,B) The comparisons of serum lipid composition between C57BL/6 (B6) *Apoe*^−/−^ and NOD *Apoe*^−/−^, B6 *Apoe*^−/−^ and NOD *Ldlr*^−/−^ animals. Blood cholesterol (TC), triglyceride (TG), low-density lipoprotein (LDL) and high-density lipoprotein (HDL) concentrations were measured in two independent experiments, and animals were fed a HFD for 12 weeks. NOD *Apoe*^−/−^ mice, *n*=4; NOD *Ldlr*^−/−^ mice, *n*=5; B6 *Apoe*^−/−^ mice, *n*=9. (C) *En face* micrographs of mounted aortas stained with Oil Red O (red). Animals were fed a HFD for 12 weeks (SMZ745, Nikon, 0.335×). (D) Quantitation of plaque areas relative to the area of the aorta from C in two independent experiments. NOD *Apoe*^−/−^ mice, *n*=4 (male=2, female=2); NOD *Ldlr*^−/−^ mice, *n*=5 (male=3, female=2); B6 *Apoe*^−/−^ mice, *n*=9 (male=4, female=5). Statistics by two-tailed, unpaired Student's *t*-test: *P<0.05, **P<0.01, ***P<0.001, ns, not significant.

The ApoE- and LDLR-deficient mouse models have been extensively used to study hyperlipidemia and atherosclerosis (Kennedy et al., 2010). When placed on a HFD, *Ldlr*^−/−^ mice develop severe hyperlipidemia and extensive atherosclerosis (Ishibashi et al., 1993), and *Apoe*^−/−^ mice are also frequently used to model hypercholesterolemia and severe atherosclerosis (Plump et al., 1992). However, such models have not been applied on the pure NOD background. We first compared lipid levels and atherosclerosis development in the NOD mice that were deficient in single genes, by analyzing ApoE- or LDLR-deficient NOD mice, to controls (ApoE-deficient C57BL/6 mice; hereafter referred to as B6 *Apoe*^−/−^ mice). Mice were fed a HFD for 12 weeks, and we found that, in comparison to B6 *Apoe*^−/−^ mice, NOD *Apoe*^−/−^ mice had significantly lower total cholesterol (TC) and low-density lipoprotein (LDL) level in serum, while the NOD *Ldlr*^−/−^ animals had higher triglyceride (TG) and LDL levels in serum, and both of the NOD mutants had higher high-density lipoprotein (HDL) levels than B6 *Apoe*^−/−^ mice (Fig. 2A,B). In addition, aortic sinus sections were compared by Oil Red O (Fig. 2A,B, red) staining to visualize lesion area and obstruction. As shown in Fig. 2C,D, the NOD *Apoe*^−/−^ and NOD *Ldlr*^−/−^ mice did not show a difference in atherosclerosis development and the narrowing area by plaques was not obvious (2.3% for both mutants), and B6 *Apoe*^−/−^ mice had 7-fold more plaque formation in the lesion area (16.2%). The sections of aortic sinus hematoxylin and eosin (H&E) staining from three types of mice showed that B6 *Apoe*^−/−^ had more severe atherosclerosis than NOD *Apoe*^−/−^ and NOD *Ldlr*^−/−^ mice. However, the resistance phenotype was less dramatic in the sections of aortic sinus than that observed in *en face* analyses of aorta (Fig. S2A-D). In further analyses, the correlation between plaque area and serum TC level was assessed in the three groups: B6 *Apoe*^−/−^, NOD *Apoe*^−/−^ and NOD *Ldlr*^−/−^ mice (Fig. S2E). Such data indicated that the NOD background had a significant impact on lipid metabolisms, as NOD single-deficient models had lower levels of atherogenic lipids and higher athero-protective HDLs than B6 *Apoe*^−/−^ mice. Our data from single-gene-deficient NOD *Apoe*^−/−^ and NOD *Ldlr*^−/−^ mutants confirmed that the NOD genetic background was highly resistant to atherosclerosis development.

### Double-knockout NOD *Apoe*^−/−^*Ldlr*^−/−^ mice develop severe atherosclerosis and hyperlipidemia

**Fig. 3.**
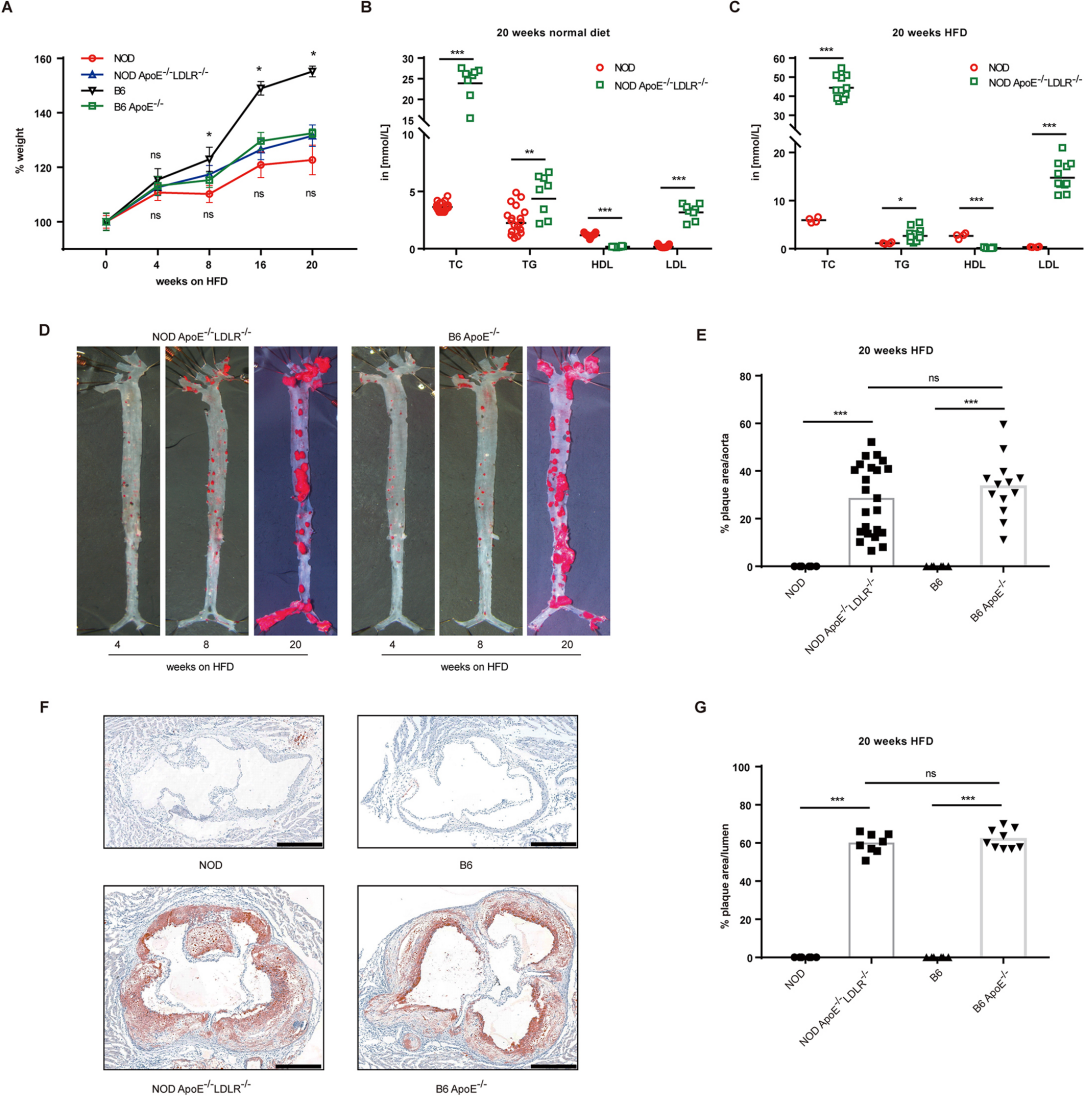
**Hyperlipidemia and atherosclerosis in double-knockout NOD** ***Apoe*^−/−^*Ldlr*^−/−^ mice.** (A) Kinetics of body weight of NOD *Apoe*^−/−^*Ldlr*^−/−^ and B6 *Apoe*^−/−^ mice fed a HFD for 20 weeks, with NOD and B6 wild-type controls. For each time point, at least three animals were analyzed for each genotype (*n*=3-55). (B) Blood cholesterol (TC), triglyceride (TG), high-density lipoprotein (HDL) and low-density lipoprotein (LDL) concentrations of mice on normal diet for 20 weeks analyzed in two independent experiments involving NOD *Apoe*^−/−^*Ldlr*^−/−^ and NOD control mice (*n*=8-19). ‘in [mmol/L]’, the levels of TC, TG, LDL and HDL were measured in mmol/l. (C) Blood TC, TG, HDL and LDL concentrations of mice on a HFD for 20 weeks analyzed in two independent experiments involving NOD *Apoe*^−/−^*Ldlr*^−/−^ and NOD mice (*n*=4-11). (D) Representative images of Oil-Red-O-stained aortas of mice on a HFD for 4, 8 and 20 weeks (*en face* assay). (E) Quantitative analysis of *en face* lesion area of NOD *Apoe*^−/−^*Ldlr*^−/−^ and B6 *Apoe*^−/−^ mice fed a HFD for 20 weeks, with NOD and B6 wild-type control mice [NOD, *n*=6 (male=3, female=3); NOD *Apoe*^−/−^*Ldlr*^−/−^, *n*=23 (male=10, female=13); B6, *n*=6 (male=3, female=3); B6 *Apoe*^−/−^, *n*=13 (male=4, female=9)]. Data were collected from two independent experiments with the same animals used in D (*n*=6-23). (F) Four representative microscopy images of aortic root sections from NOD *Apoe*^−/−^*Ldlr*^−/−^ and B6 *Apoe*^−/−^ mice placed on a HFD for 20 weeks, with controls of NOD and B6 wild-type mice. Scale bars: 400 μm. (G) Quantitation of plaque area relative to the area of the aortic lumen from F [NOD, *n*=6 (male=3, female=3); NOD *Apoe*^−/−^*Ldlr*^−/−^, *n*=8 (male=5, female=3); B6, *n*=6 (male=3, female=3); B6 *Apoe*^−/−^, *n*=9 (male=4, female=5)] (*n*=6-9). Statistics by two-tailed, unpaired Student's *t*-test: *P<0.05, **P<0.01, ***P<0.001, ns, not significant.

On the C57BL/6 background, *Apoe*^−/−^*Ldlr*^−/−^ double-knockout mice have cholesterol and lipoprotein profiles similar to the *Apoe* single-mutant mice, and reportedly develop atherosclerotic plaques even more rapidly than *Apoe* single-knockout mice (Ishibashi et al., 1994). To investigate whether severe atherosclerosis could be developed on the NOD background, we therefore further analyzed NOD mice deficient in both ApoE and LDLR. We selected mice from F2 progeny that were derived from sequencing-validated F0 and F1 animals. The selected NOD *Apoe*^−/−^*Ldlr*^−/−^ mice were fed with a HFD for 20 weeks and the body weight was documented on a 4-week basis; the lipid levels as well as pathological development were analyzed by the end of 20 weeks. Four groups of animals – NOD *Apoe*^−/−^*Ldlr*^−/−^ mice, NOD control mice, B6 *Apoe*^−/−^ mice and B6 control mice – were fed a HFD and all of the groups steadily gained body weight. The body weight of B6 *Apoe*^−/−^ mice fed a HFD increased significantly less than the B6 controls, and such a trend was enlarged until the end of the experiment, which was consistent with previous studies (Bartelt et al., 2011). However, strikingly, the NOD *Apoe*^−/−^*Ldlr*^−/−^ mice did not show such difference in body weight from the NOD control mice throughout the experiment (Fig. 3A). We also analyzed food intake on a weekly basis between the four groups of animals, and the results showed that NOD *Apoe*^−/−^*Ldlr*^−/−^ mice had no difference in intake of food from NOD wild-type mice, and the same results were observed between B6 *Apoe*^−/−^ mice and B6 wild-type mice (Fig. S3A,B).

Based on our previous observation with the single-gene-deficient NOD models (which had lower atherogenic lipids and higher athero-protective HDL, and, more importantly, were not developing obvious atherosclerosis as found in B6 *Apoe*^−/−^ mice), we compared lipid levels in the NOD *Apoe*^−/−^*Ldlr*^−/−^ mice in a more intensive manner by analyzing lipids from mice on both chow food and a HFD. In mice fed with normal diet for 20 weeks, we analyzed the blood lipids and found that NOD *Apoe*^−/−^*Ldlr*^−/−^ mice had significantly higher levels of TC, TG and LDL, and a significantly lower level of HDL, than the NOD mice (Fig. 3B). When comparing NOD *Apoe*^−/−^*Ldlr*^−/−^ mice and NOD mice following 20 weeks of HFD, hyperlipidemia was more severe in NOD *Apoe*^−/−^*Ldlr*^−/−^ mice as TC, TG and LDL were all significantly higher than in NOD mice (Fig. 3C). Additional data plotting of hyperlipidemia in NOD *Apoe*^−/−^*Ldlr*^−/−^ mice and NOD mice on a HFD for 4 and 8 weeks showed that TG was not significantly different, but TC and LDL was 9-fold and 30-fold lower in the control mice, respectively, and HDL was 30-fold higher in control animals (Fig. S3C). In parallel, we compared the blood lipids between B6 controls and B6 *Apoe*^−/−^ mice after 4, 8 and 20 weeks of being fed a HFD, and the trends of differences were like the results between NOD and NOD *Apoe*^−/−^*Ldlr*^−/−^ mutants (Fig. S3E). Interestingly, B6 and NOD wild-type mice did not show lipid level differences after 20 weeks on a HFD (data not shown); however, NOD *Apoe*^−/−^*Ldlr*^−/−^ had significantly higher levels of TC, TG and LDL and lower HDL than the B6 *Apoe*^−/−^ mice (Fig. S3D). In further experiments, we compared the pathology of global aorta and aortic sinus sections, and found that both NOD *Apoe*^−/−^*Ldlr*^−/−^ and B6 *Apoe*^−/−^ mice develop severe atherosclerosis and that the percentage of plaque area did not differ (Fig. 3D-G, Fig. S3F-I). However, when analyzed separately, the NOD *Apoe*^−/−^*Ldlr*^−/−^ female mice had less atherosclerosis plaque area than the B6 *Apoe*^−/−^ mice, whereas the male mice were not different (Fig. S3J,K). In addition, significant differences were observed between male and female animals in the NOD *Apoe*^−/−^*Ldlr*^−/−^ mice, with the male mice developing more severe atherosclerosis (data not shown).

Our data suggest that the NOD background had an impact on lipid metabolism and that NOD mice are more resistant to developing hyperlipidemia; however, simultaneous genetic inactivation of both ApoE and LDLR was sufficient to model both severe hyperlipidemia and severe atherosclerosis. The data also suggest that more severe hyperlipidemia was necessary to model atherosclerosis on the NOD background, as single-gene knockout of ApoE and LDLR on NOD was not sufficient to cause obvious atherosclerosis.

### Destruction of pancreatic islets concomitant with decreased hyperglycemia in NOD *Apoe*^−/−^*Ldlr*^−/−^ mice

**Fig. 4.**
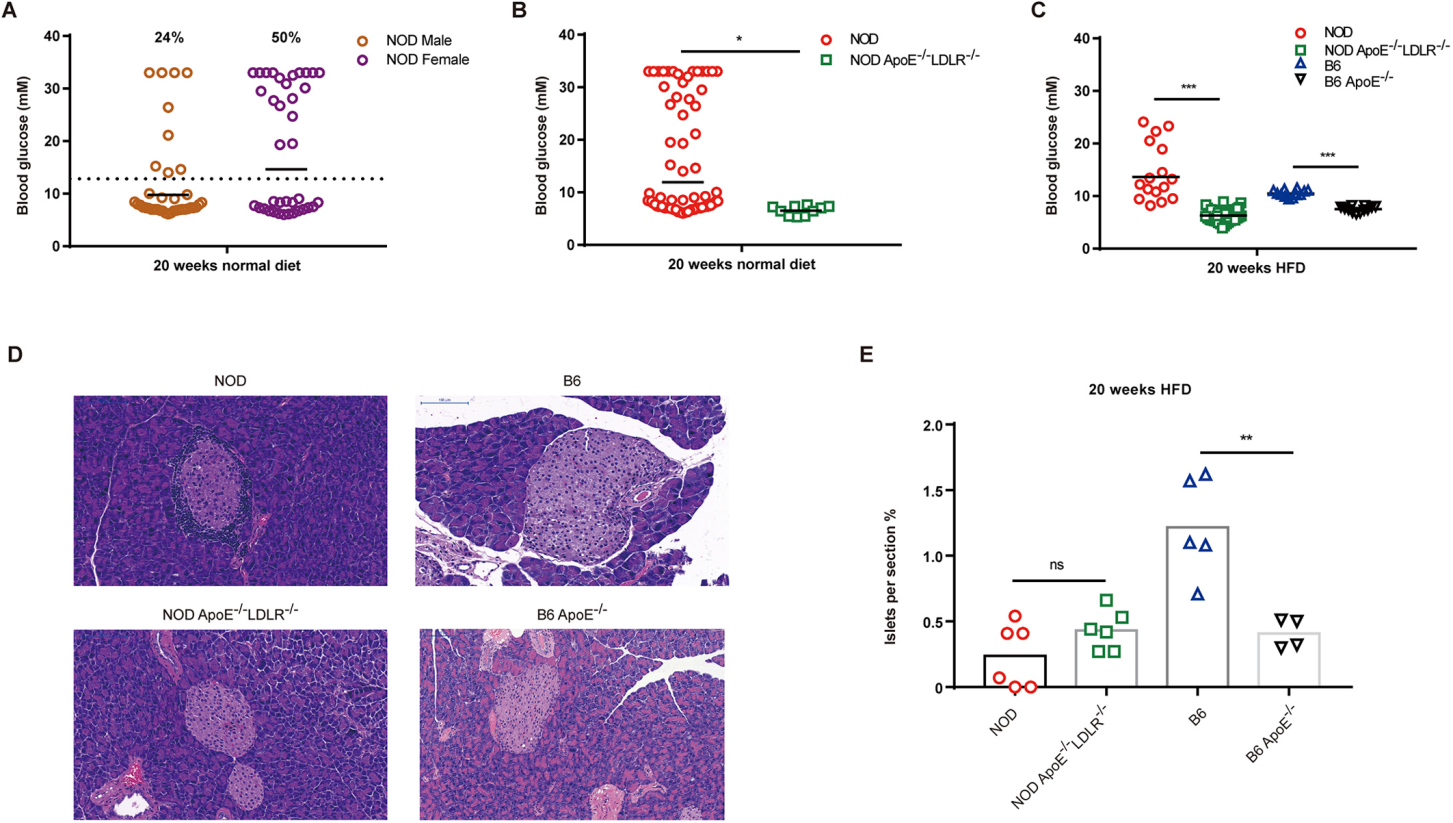
**Blood glucose and islet size in NOD** ***Apoe*^−/−^*Ldlr*^−/−^ mice on a normal and** **HFD.** (A) The blood glucose levels and incidence of type 1 diabetes (T1D) in male and female NOD mice after 20 weeks of normal diet with a diagnostic threshold of 14 mM. A total of 24% of male and 50% of female NOD mice develop spontaneous T1D (*n*=38). (B) Blood glucose levels in NOD and NOD *Apoe*^−/−^*Ldlr*^−/−^ animals after 20 weeks of normal diet (*n*=10-76). (C) Blood glucose levels in NOD *Apoe*^−/−^*Ldlr*^−/−^ and B6 *Apoe*^−/−^ animals after 20 weeks of HFD, with controls of NOD and B6 mice (*n*=12-42). (D) Histologic analyses of pancreas from NOD, NOD *Apoe*^−/−^*Ldlr*^−/−^, B6 and B6 *Apoe*^−/−^ mice fed with 20 weeks of HFD. Scale bars: 100 μm. (E) Islet size quantitation in H&E-stained pancreas sections shown as percentage of total pancreas area scanned in each section. For each animal, one section was selected for analysis (NOD, *n*=6; NOD *Apoe*^−/−^*Ldlr*^−/−^, *n*=6; B6, *n*=5; B6 *Apoe*^−/−^, *n*=4) (*n*=4-6). Statistics by two-tailed, unpaired Student's *t*-test: *P<0.05, **P<0.01, ***P<0.001, ns, not significant.

NOD mice develop autoimmunity in T-cell-dependent manners, which cause pathological consequences affecting multiple organs, including the pancreas (Anderson and Bluestone, 2005; D'Alise et al., 2008). Destruction of autoreactive T cells in islets could be manifested as spontaneous hyperglycemia and T1D, the incidence of which could vary between facilities (Anderson and Bluestone, 2005). We first analyzed the incidence of T1D in NOD wild-type mice by setting the diagnostic threshold at 14 mM blood glucose (Lum et al., 1991) and randomly measuring blood glucose without fasting, and we found that 24% of males versus 50% of female animals had T1D, consistent with previous reports (Fig. 4A). We analyzed the blood glucose levels in NOD *Apoe*^−/−^*Ldlr*^−/−^ mice with normal diet or HFD for 20 weeks from the same housing condition. In our experiments, on normal diet, NOD *Apoe*^−/−^*Ldlr*^−/−^ mice had a decreased level of blood glucose compared with the NOD wild-type mice (Fig. 4B). In the mice fed a HFD, both NOD *Apoe*^−/−^*Ldlr*^−/−^ and B6 *Apoe*^−/−^ had significantly lower levels of blood glucose than their wild-type controls (Fig. 4C). Such trends existed in mice fed a HFD for 4–8 weeks (Fig. S4A,B). Using pancreas sections and H&E staining, we evaluated the islet distribution and found that, after 20 weeks of HFD, B6 *Apoe*^−/−^ mice had significantly less dense islets than the B6 wild-type mice, whereas both NOD *Apoe*^−/−^*Ldlr*^−/−^ mice and NOD wild-type controls had similar atrophic islets (Fig. 4D,E). In further experiments, white blood cell infiltration and apoptosis were analyzed in the pancreas of both NOD and NOD *Apoe*^−/−^*Ldlr*^−/−^ mice. We found that both groups had obvious inflammatory cell infiltration as shown by CD45-positive staining. Such results suggest that both NOD and NOD *Apoe*^−/−^*Ldlr*^−/−^ mice had pancreas inflammation (Fig. S4C). Since NOD *Apoe*^−/−^*Ldlr*^−/−^ mice had lower blood glucose levels than NOD mice on a HFD, we analyzed insulin levels in B6, NOD and NOD *Apoe*^−/−^*Ldlr*^−/−^ mice aged 20 weeks on normal diet; as expected, both NOD and NOD *Apoe*^−/−^*Ldlr*^−/−^ mice had significantly lower insulin levels than B6 mice (Fig. S4D). However, NOD mice were found to have significantly lower insulin levels than NOD *Apoe*^−/−^*Ldlr*^−/−^ 20-week-old mice, which correlated with the lower blood glucose level observed in NOD *Apoe*^−/−^*Ldlr*^−/−^ animals (Fig. S4D). To analyze apoptosis in the pancreas, caspase 3 was stained in NOD and NOD *Apoe*^−/−^*Ldlr*^−/−^ mice. We noticed caspase-3-positive staining in both groups of animals; however, NOD *Apoe*^−/−^*Ldlr*^−/−^ mice had less intensive caspase-3 staining, which could explain the higher level of insulin in NOD *Apoe*^−/−^*Ldlr*^−/−^ mice than NOD wild-type controls (Fig. S4C).

Our results indicated that B6 wild-type mice had a more intact islet structure than B6 *Apoe*^−/−^ mice after 20 weeks on a HFD; however, both NOD and NOD *Apoe*^−/−^*Ldlr*^−/−^ mice had a similar destructed islet structure (Fig. 4D,E). The data showed that, in comparison to NOD wild-type controls, NOD *Apoe*^−/−^*Ldlr*^−/−^ mice had decreased hyperglycemia when fed a HFD, which was similarly documented in a previous study involving B6 controls and B6 *Apoe*^−/−^ mice (Bartelt et al., 2011). To understand whether such differences of blood glucose between NOD wild-type controls and NOD *Apoe*^−/−^*Ldlr*^−/−^ mice on a HFD could be caused by the ability of the animals to metabolize glucose, mice on a HFD for 8 weeks were subjected to a glucose tolerance test, and blood glucose of NOD wild-type and NOD *Apoe*^−/−^*Ldlr*^−/−^ mice that had undergone overnight fasting were measured 15, 30, 60 and 120 min post-intraperitoneal injection of 2 g/kg total body weight glucose. We did not observe a significant difference between NOD control mice and their mutant counterparts for blood glucose (Fig. S4E,F). However, *in vivo* functional analysis of pancreas by measuring the insulin induction in NOD *Apoe*^−/−^*Ldlr*^−/−^ mice and NOD controls showed that, in 20-week-old animals, NOD mice did not have elevated insulin in blood, whereas the double-knockout mice had induced insulin secretion (Fig. S4G,H). Taken together, our data showed that both NOD and NOD *Apoe*^−/−^*Ldlr*^−/−^ mice had destructed pancreatic islets with inflammatory infiltration, although less intensive caspase-3 staining in NOD *Apoe*^−/−^*Ldlr*^−/−^ mice might suggest that less severe apoptosis could contribute residual insulin-secreting cells and a higher level of insulin.

### Inflammatory response of NOD *Apoe*^−/−^*Ldlr*^−/−^ mice to hyperlipidemia

**Fig. 5.**
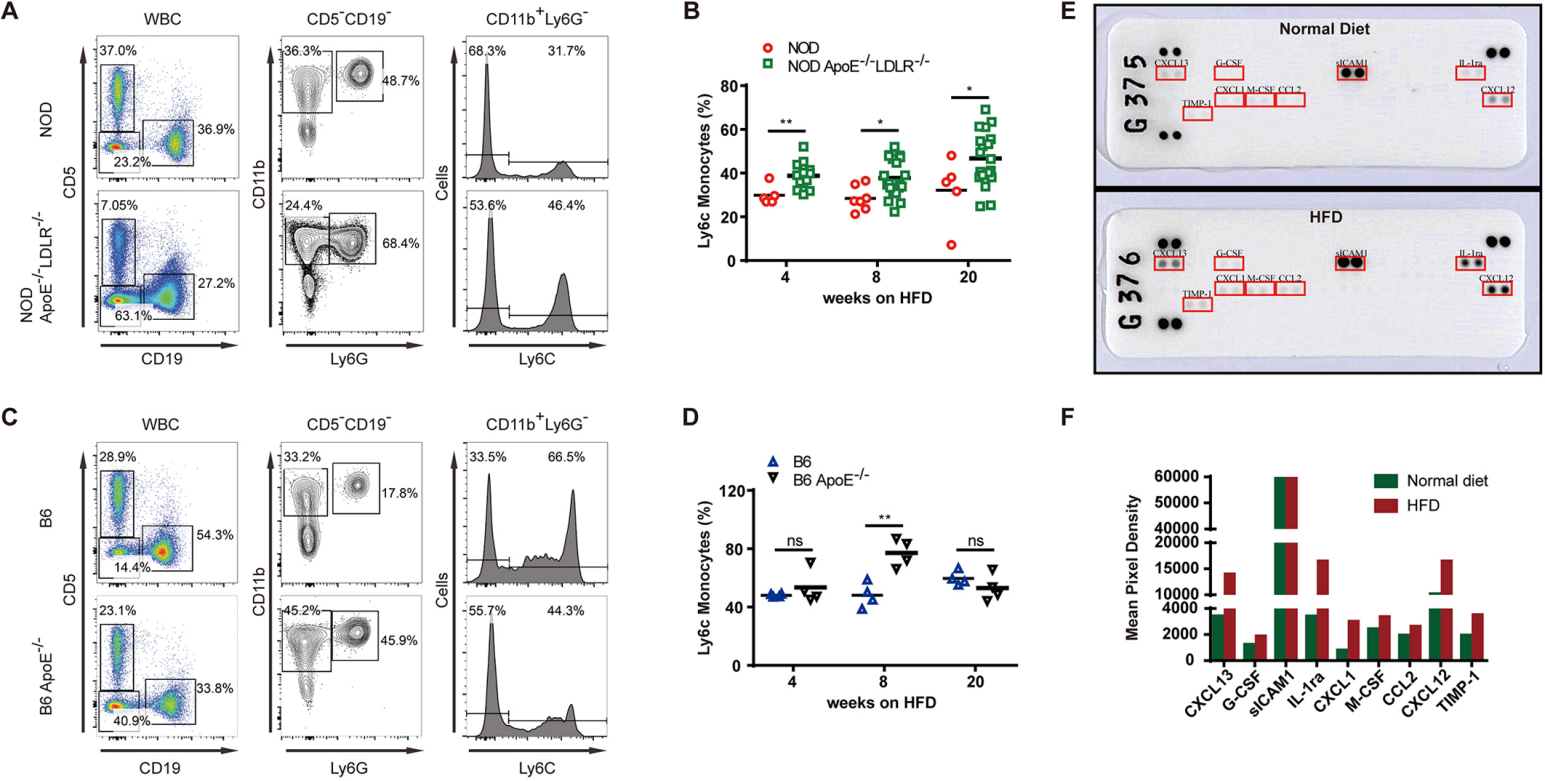
**Immune response of NOD** ***Apoe*^−/−^*Ldlr*^−/−^ mice to hyperlipidemia.** (A) The gating for Ly6C-positive monocytes in the splenocytes of NOD *Apoe*^−/−^*Ldlr*^−/−^ mice, with NOD as control (*n*=5-20). (B) Frequency of Ly6C-positive monocytes in NOD and NOD *Apoe*^−/−^*Ldlr*^−/−^ mice. (C) The gating for Ly6C-positive monocytes in the splenocytes of B6 *Apoe*^−/−^ and B6 control (*n*=4). (D) Frequency of Ly6C-positive monocytes in B6 and B6 *Apoe*^−/−^ mice. (E) Serum cytokines in NOD *Apoe*^−/−^*Ldlr*^−/−^ mice fed a HFD or normal diet for 20 weeks. Serum samples (100 μl) were collected from three mice from each group and analyzed by the Proteome Profiler Mouse Cytokine Array Kit. Data shown are from a 5-min exposure. (F) Quantification of the cytokine expression in samples used in E. Statistics by two-tailed, unpaired Student's *t*-test: *P<0.05, **P<0.01, ns, not significant.

Atherosclerosis is defined as a chronic inflammatory disorder with involvement of various cellular and molecular components, including macrophages, T cells and cytokines (Ross, 1999; Gotsman et al., 2008). We performed immunophenotyping to investigate monocytes and T cells in spleen of NOD *Apoe*^−/−^*Ldlr*^−/−^ mice and B6 *Apoe*^−/−^ mice that were fed a HFD for 20 weeks, with comparison to NOD and B6 wild-type controls. Regulatory T cells (Tregs) maintaining immune tolerance can be induced by an inflammatory response to hyperlipidemia (Pettersson et al., 2012). We observed that NOD *Apoe*^−/−^*Ldlr*^−/−^ mice had a significantly higher frequency of Tregs in splenocytes than wild-type controls after 20 weeks of HFD (Fig. S5A,B). In the same experiments, B6 *Apoe*^−/−^ mice and B6 wild-type controls were also compared for Treg response, and we found that the Treg frequency increase was conserved between the NOD and B6 backgrounds (Fig. S5C,D). In spleen monocytes, we found that NOD *Apoe*^−/−^*Ldlr*^−/−^ mice, when fed a HFD, displayed an increase in Ly6C^high^ pro-inflammatory population in frequencies, significantly higher than NOD controls throughout 4-20 weeks on a HFD (Fig. 5A,B). Interestingly, the frequencies of pro-inflammatory monocytes were distributed differently on the B6 background, with an increase of the Ly6C^high^ pro-inflammatory population of B6 *Apoe*^−/−^ mice only in the group fed a HFD for 8 weeks (Fig. 5C,D). To further analyze the immune cells in aortic plaques, we prepared cells from aorta from NOD *Apoe*^−/−^*Ldlr*^−/−^ mice and B6 *Apoe*^−/−^ mice. CD45 labeling was used to set the threshold for FACS acquisition and Sytox Blue staining was used to exclude dead cells (Fig. S6A,B). We did not find a significant difference between T-cell activation as measured by CD44 mean fluorescence intensity and Treg frequencies (Fig. S6C,D). However, NOD *Apoe*^*–/–*^*Ldlr*^–/–^ mice had significantly higher frequencies of CD11b^+^F4/80^+^ tissue macrophages, in which Ly6C expression did not display a difference in such double-knockout mice (Fig. S6E,F).

As cytokines and chemokines are implicated in atherosclerosis development, we performed protein array analyses of the NOD *Apoe*^−/−^*Ldlr*^−/−^ model in cytokine production (Kleemann et al., 2008; Zernecke and Weber, 2010). The serum cytokines were analyzed by a mouse cytokine array kit that included 40 types of cytokines, and our results showed that NOD *Apoe*^−/−^*Ldlr*^−/−^ mice on 20 weeks of HFD had nine increased serum cytokines/chemokines when compared to NOD *Apoe*^−/−^*Ldlr*^−/−^ mice on normal diet. Among them CXCL13, IL-1Ra and CXCL12 were abundant and had a magnitude of increase over 5-fold (Fig. 5E,F). In further experiments, we performed such comparisons between NOD *Apoe*^−/−^*Ldlr*^−/−^ mice and B6 *Apoe*^−/−^ mice: CXCL13 and IL-1Ra levels were 3-fold higher in NOD *Apoe*^−/−^*Ldlr*^−/−^ mice, suggesting that these two cytokines could be preferentially produced under the NOD background (Fig. S5E,F). CXCL13 was found to be implicated in plaque stability in humans (Smedbakken et al., 2012). IL-1Ra has been documented to have anti-atherosclerotic roles, as IL-1Ra knockout mice on an ApoE-deficient background develop more severe atherosclerosis (Isoda et al., 2004). Analyses of immunophenotyping showed an increase of frequencies in Tregs and Ly6C^high^ pro-inflammatory monocytes in NOD *Apoe*^−/−^*Ldlr*^−/−^ mice, and such responses were found to be generally conserved on the B6 background even though the elevation of Ly6C^high^ pro-inflammatory monocytes on the B6 background were more time dependent. Our data also revealed that CXCL13 and IL-1Ra were more induced on the NOD background by hyperlipidemia.

## DISCUSSION

Inbred mouse strains harbor a large number of genetic polymorphisms that genetically condition their susceptibility or resistance to atherosclerosis, and studies in murine models could provide fundamental knowledge required to understand the human pathology (Paigen et al., 1985; Doran et al., 2016). The NOD strain has been well documented as a model to study autoimmunity and its associated diseases, such as T1D (Pearson et al., 2016). Even though atherosclerosis in complication with autoimmunity has triggered interest, a genetic model on the pure NOD background to model atherosclerosis has not been reported. In this study, we performed CRISPR/Cas9 genome editing in the fertilized oocytes of NOD mice. Following a HFD for 12 weeks, we observed high resistance to atherosclerosis of mice deficient in either ApoE or LDLR, the key molecules for lipid metabolism and broadly used in atherosclerotic models. Therefore, we validated the resistance of the NOD genome to atherosclerosis in comparison to the susceptible C57BL/6 mice.

From a genetics perspective, such single-gene-deficient mice could be valuable to construct pedigree to map genetic factors that determine the susceptibility of C57BL/6 mice to atherosclerosis. It is important to note that non-redundant genetic polymorphisms of NOD mice could benefit identification of novel genes, as there are 4-million SNPs between NOD and B6 mice, and 143,489 of such SNPs are private to the NOD genome (Keane et al., 2011). Interestingly, another resistant strain to atherosclerosis, C3H/HeJ, was found to possess 8-fold less such private SNPs among the inbred lines sequenced (Tabibiazar et al., 2005; Keane et al., 2011). The genetic diversity of NOD gives rise to a valuable tool for understanding the molecular mechanisms underlying resistance to atherosclerosis.

We further established the NOD mice deficient in both ApoE and LDLR. Interestingly, in the double-knockout mice, we found that such animals developed comparable atherosclerosis as ApoE-deficient B6 mice. In addition, we found that the lipid composition differences existed between NOD *Apoe*^−/−^*Ldlr*^−/−^ mice and B6 *Apoe*^−/−^ mice in spite of the fact that their susceptibility to atherosclerosis induced by a HFD was similar. Notably, the double-knockout NOD mice did not show any difference from their wild-type control for gain of body weight when placed on a HFD, which was dramatically different for B6 *Apoe*^−/−^ mice. From a metabolic perspective, it is also interesting to compare the lipids in serum between B6 *Apoe*^−/−^ mice and NOD *Apoe*^−/−^ mice, and we found that the NOD background showed less TC and LDL than the B6 counterpart. ApoE and LDLR deficiency on the B6 background itself had different pathological features, as reviewed in the literature (Getz and Reardon, 2016); however, strikingly, we found that NOD *Apoe*^−/−^ and NOD *Ldlr*^−/−^ mice were both highly resistant to atherosclerosis, with 7-fold fewer plaques than the B6 *Apoe*^−/−^ mice (Getz and Reardon, 2012).

Our data suggest that the NOD background could impact lipid metabolism and confer resistance to hyperlipidemia development; however, simultaneous genetic inactivation of both ApoE and LDLR was sufficient to model both severe hyperlipidemia and severe atherosclerosis. In the double-knockout NOD *Apoe*^−/−^*Ldlr*^−/−^ mice, it still remained intriguing that these animals had obvious destructed islets compared with the NOD mice, which had inflammatory infiltration characterized by auto-reactive T cells, but hyperglycemia was at least partially protected. Less intensive caspase-3 staining in the double-knockout mice in comparison to the NOD controls might suggest that less severe apoptosis could contribute residual insulin-secreting cells and a higher level of insulin in NOD *Apoe*^−/−^*Ldlr*^−/−^ mice. Indeed, the pancreatic functional assay showed a protective role of the double gene deficiency in insulin secretion on the NOD background.

The study of atherosclerosis in the setting of autoimmunity or in complication with diabetes is necessary because such a condition occurs in the clinic; however, mice modeling atherosclerosis complicated with diabetes have still not been developed by genetic means on the NOD background (Eckel and Eisenbarth, 2012; Mitchell, 2012). Here, we provided experimental data that NOD mice that were simultaneously deficient in both ApoE and LDLR developed severe aorta occlusion, with an over 60% narrowing in aortic sinus sections. Interestingly, we found that, despite the diverged genomes between NOD and C57BL/6 mice, the T-cell response to hyperlipidemia was quite conserved in terms of an increase in frequency of regulatory T cells in mice fed a HFD. We further profiled in the double-knockout NOD mice the cytokines and chemokines implicated in atherosclerosis, and found that CXCL13 and IL-1Ra were more induced on a HFD compared with normal diet. Such differences in cytokines and chemokines could be further investigated for their roles in the susceptibility to atherosclerosis. Such observations also suggest that NOD *Apoe*^−/−^*Ldlr*^−/−^ mice could be different from B6 *Apoe*^−/−^ animals in genetic etiology even though their severity of HFD-induced atherosclerosis was comparable. We observed in the double-knockout model that the blood glucose level was lower than in ApoE-deficient C57BL/6 mice. The mechanisms to explain why the blood glucose level was lower in NOD *Apoe*^−/−^*Ldlr*^−/−^ mice while their islets were severely destructed as much as in the NOD wild-type mice remain to be studied. We analyzed the LDL-C level between *Apoe* single-gene-knockout and *Apoe*^−/−^*Ldlr*^−/−^ double-gene-knockout animals on a HFD, and found that the LDL-C level was significantly higher in double-knockout animals, suggesting that lower LDL-C could contribute to resistance of single-gene *Apoe*-knockout mice to atherosclerosis. However, more detailed analyses involving VLDL-C and IDL-C fractions could help delineate the pathological consequence that the NOD background confers on disease resistance.

In this model, NOD *Apoe*^−/−^*Ldlr*^−/−^ mice were established in complete absence of passenger DNA and could be more optimal than genetically modified congenic mice (Vanden Berghe et al., 2015). Therefore, we developed a novel atherosclerosis mouse model on the pure NOD background that could be useful for both genetic studies of identifying novel molecules as well as a tool for investigating atherosclerosis with the complication of autoimmunity.

## MATERIALS AND METHODS

### Animals

8-week-old female NOD mice and 10-week-old male NOD mice were purchased from the Model Animal Research Center of Nanjing University, China. 10-week old male and female ICR outbred foster mice and 6- to 8-week-old C57BL/6 mice on wild-type and ApoE-deficient backgrounds were purchased from Beijing Vital River Laboratory Animal Technology Co., Ltd., and all animal procedures were performed according to guidelines approved by the committee on animal care at Xinxiang Medical University, China. The body weight and food intake were recorded on a weekly basis.

### Design of targeting site for CRISPR/Cas9 and preparation of *Cas9* mRNA and sgRNA

The genomic DNA sequence of the selected gene fragment can be submitted to an online design tool (http://crispor.tefor.net/crispor.cgi) to generate information about potential targets. Linearized plasmid pT7-3×Flag-hCas9 by *Pme*I digestion was used as template for *in vitro* transcription of *Cas9* mRNA, and was a gift from Junjiu Huang (Liang et al., 2015). For each guide sequence, a specific forward primer and a universal Scaffold-Rev primer were used for constructing T7-sgRNA-scaffold as previously described (Shao et al., 2014). The PCR products were purified and served as template for *in vitro* transcription of sgRNAs using MEGAshortscript™ Kit (Thermo Fisher Scientific, AM1354).

### Microinjection of zygotes and embryo transfer

*Cas9* mRNA (50 ng/μl) and sgRNA (50 ng/μl) were microinjected into the cytoplasm of fertilized eggs in M2 medium (Sigma) by using a standard microinjection system (Eppendorf TransferMan^®^ 4r, Eppendorf, Germany; Nikon Ti, Nikon, Japan). Injected eggs were washed in M2 medium three times and then cultured in 50 μl of M16 medium covered with embryo-tested mineral oil (Sigma) at 37°C in 5% CO_2_ overnight to the two-cell stage. Two-cell embryos injected with *Cas9* mRNA and sgRNA were transferred into the oviductal ampullas (10-20 embryos per oviduct) of 10-week-old female ICR mice that were mated with vasectomized ICR males the previous night.

### Genotyping of CRISPR/Cas9-induced *Apoe* and *Ldlr* mutations by fluorescence PCR and immunoblotting

Genomic DNA samples were prepared from F0 newborn tails by a standard extraction method. DNA extracts were subjected to PCR analysis using Phusion High-Fidelity DNA Polymerase (Thermo Fisher Scientific) and 5′-fluorescein-amidite (FAM)-labeled or 5′-hexachloro-fluorescein (HEX)-labeled primers (Table S1). The PCR amplicons were resolved using an ABI 3730 DNA analyzer. Data analysis was performed by GeneMapper software v3.1. The positions and sizes of the peaks indicate the lengths and relative amounts of PCR products (Velasco et al., 2007). For sequencing, PCR products were further cloned with pGM-Simple-T Fast Kit (Tiangen). In general, ten colonies were picked up from each agar plate and were determined by Sanger sequencing. The liver tissues were lysed with RIPA buffer (Beyotime, China) and submitted to western blotting using anti-LDL receptor antibody (ab5281) and anti-apolipoprotein E antibody (ab183597).

### Atherosclerosis modeling and histological analysis

8-week-old male or female mice were fed a HFD (D12108C, 40% fat, 1.25% cholesterol, Research Diets, Inc.) for another 4, 8, 12, 16 or 20 weeks. Mice were euthanized, and the heart and aorta were harvested for measuring lesions at designated time points. Hearts embedded in OCT were sectioned through the aortic root (10 μm) and stained with hematoxylin and eosin (H&E) or Oil Red O for lesion quantification. Images were acquired on a Pannoramic MIDI II (3D HISTECH). The quantification of *en face* lesion was performed by removing surrounding fat tissue of the aorta with forceps under a dissection microscope, and the inner surface of the aorta, including aortic arch, thoracic and abdominal aorta, was exposed. Aortas were stained with Oil Red O in 70% ethanol for 90 min at room temperature followed by 5 min destain in 70% ethanol as described (Warnatsch et al., 2015). Lesion area was presented as a percentage of the total area of the aorta. Serum samples were from retro-orbital bleeding of mice under anesthesia by isoflurane for analyses of lipids. Homogeneous enzymatic colorimetric assays were used to measure serum HDL-C (HDL; High Density Lipoprotein Cholesterol Assay Kit, B00311, Lepu Diagnostics Company, Beijing) and LDL-C (LDL; Low Density Lipoprotein Cholesterol Assay Kit, B00411, Lepu Diagnostics Company, Beijing) levels, and enzymatic colorimetric assays were used to measure triglycerides (TG; Triglycerides Assay Kit, B00101, Lepu Diagnostics Company, Beijing) and total cholesterol (TC; Cholesterol Assay Kit, B00201, Lepu Diagnostics Company, Beijing) levels by the ADVIA 2400 analyzer (SIEMENS). The serum samples were used to determine the cytokine levels by mouse cytokine array panel A (R&D Systems, ARY006).

Pancreas samples were dissected from mice fed a HFD for 20 weeks and fixed with 4% paraformaldehyde/phosphate-buffered saline solution overnight. Paraffin-embedded pancreatic tissues fixed in 4% paraformaldehyde (HistoLab Products) were sectioned (10 μm). Islet areas, reported as percentage of total pancreas area, were analyzed from H&E-stained sections and quantified with ImageJ software; for each animal, one section was selected for analysis. The tissue sections were incubated for 24 h with antibodies against CD45 and caspase 3 following the manufacturer's recommendations (Servicebio, China). The immunoreactivity was visualized with 3,3′-diaminobenzidine (DAB) color reaction and counterstained with hematoxylin. Blood glucose levels were monitored using a Reflotron Plus diagnostic machine and test sticks (Roche Diagnostics, Germany). Serum insulin levels were measured using the Rat/Mouse Insulin ELISA kit (Millipore, EZRMI-13K). Glucose tolerance test (GTT) was performed by intraperitoneal (i.p.) injection of 2 g/kg body weight glucose after overnight fasting, and blood was collected at 0, 15, 30, 60 and 120 min. Glucose-stimulated insulin secretion (GSIS) *in vivo* was analyzed by i.p. injection of glucose (2 g/kg body weight), and blood was collected at 0, 5, 15, 30 and 60 min.

### Immunophenotyping by flow cytometry

Flow cytometric analyses were performed by staining the splenocytes of mice with monoclonal antibody mixes. The antibodies used in this study are listed in Table S2. The antibody labeling experiments were done as documented in our previous studies for mouse immunophenotyping (Liang et al., 2013). In brief, for splenocytes, 1 million cells were stained in 100 µl of staining buffer with antibody mixes, and acquired on the FACSCanto flow cytometer (BD Biosciences, USA). For immunophenotyping of the aorta, mice were perfused with PBS from the left ventricle of the heart. Adipose tissues and para-aortic lymph nodes were removed before tissue dissociation and single-cell preparation. The whole aorta involved the aortic arch, ascending, descending, thoracic and abdominal portions in this study. The tissue was segmented by surgical scissor followed by digestion with 50 µg/ml Liberase DH (Roche) and 40 U/ml DNase I (NEB) for 30 min at 37°C (Butcher et al., 2011). The single cells were stained with antibody mix and analyzed by using a flow cytometer. The FACS data was analyzed using Flowjo software version 10.0.

### Statistical analysis

All data were compared between two groups and analyzed with GraphPad Prism software (version 7.0). Data are presented as means±s.e.m. Statistical significance was assessed by unpaired, two-tailed Student's *t*-test. *P*<0.05 was considered significant (*P<0.05, **P<0.01, ***P<0.001).

## Supplementary Material

Supplementary information

## References

[DMM033852C1] Anderson M. S. and Bluestone J. A. (2005). The NOD mouse: a model of immune dysregulation. *Annu. Rev. Immunol.* 23, 447-485. 10.1146/annurev.immunol.23.021704.115643 15771578

[DMM033852C2] Bartelt A., Orlando P., Mele C., Ligresti A., Toedter K., Scheja L., Heeren J. and Di Marzo V. (2011). Altered endocannabinoid signalling after a high-fat diet in Apoe(−/−) mice: relevance to adipose tissue inflammation, hepatic steatosis and insulin resistance. *Diabetologia* 54, 2900-2910. 10.1007/s00125-011-2274-6 21847582

[DMM033852C3] Bennett B. J., Davis R. C., Civelek M., Orozco L., Wu J., Qi H., Pan C., Packard R. R. S., Eskin E., Yan M. et al. (2015). Genetic architecture of atherosclerosis in mice: a systems genetics analysis of common inbred strains. *PLoS Genet.* 11, e1005711 10.1371/journal.pgen.1005711 26694027 PMC4687930

[DMM033852C4] Butcher M. J., Herre M., Ley K. and Galkina E. (2011). Flow cytometry analysis of immune cells within murine aortas. *J. Vis. Exp* 53, 2848 10.3791/2848 PMC3196167 21750492

[DMM033852C5] D'Alise A. M., Auyeung V., Feuerer M., Nishio J., Fontenot J., Benoist C. and Mathis D. (2008). The defect in T-cell regulation in NOD mice is an effect on the T-cell effectors. *Proc. Natl. Acad. Sci. USA* 105, 19857-19862. 10.1073/pnas.0810713105 19073938 PMC2604930

[DMM033852C6] Doran A. G., Wong K., Flint J., Adams D. J., Hunter K. W. and Keane T. M. (2016). Deep genome sequencing and variation analysis of 13 inbred mouse strains defines candidate phenotypic alleles, private variation and homozygous truncating mutations. *Genome Biol.* 17, 167 10.1186/s13059-016-1024-y 27480531 PMC4968449

[DMM033852C7] Eckel R. H. and Eisenbarth G. S. (2012). Autoimmune diabetes inflames the heart. *Sci. Transl. Med.* 4, 138fs118 10.1126/scitranslmed.3004219 22700952

[DMM033852C8] Getz G. S. and Reardon C. A. (2012). Animal models of atherosclerosis. *Arterioscler. Thromb. Vasc. Biol.* 32, 1104-1115. 10.1161/ATVBAHA.111.237693 22383700 PMC3331926

[DMM033852C40] Getz G. S. and Reardon C. A. (2016). Do the Apoe^–/–^ and Ldlr^–/–^ mice yield the same insight on atherogenesis? *Arterioscler. Thromb. Vasc. Biol.* 36, 1734-1741. 27386935 10.1161/ATVBAHA.116.306874 PMC5001905

[DMM033852C9] Gotsman I., Sharpe A. H. and Lichtman A. H. (2008). T-cell costimulation and coinhibition in atherosclerosis. *Circ. Res.* 103, 1220-1231. 10.1161/CIRCRESAHA.108.182428 19028921 PMC2662382

[DMM033852C10] Grainger A. T., Jones M. B., Li J., Chen M.-H., Manichaikul A. and Shi W. (2016). Genetic analysis of atherosclerosis identifies a major susceptibility locus in the major histocompatibility complex of mice. *Atherosclerosis* 254, 124-132. 10.1016/j.atherosclerosis.2016.10.011 27736672 PMC6178236

[DMM033852C11] Harel I., Benayoun B. A., Machado B., Singh P. P., Hu C.-K., Pech M. F., Valenzano D. R., Zhang E., Sharp S. C., Artandi S. E. et al. (2015). A platform for rapid exploration of aging and diseases in a naturally short-lived vertebrate. *Cell* 160, 1013-1026. 10.1016/j.cell.2015.01.038 25684364 PMC4344913

[DMM033852C12] Ishibashi S., Brown M. S., Goldstein J. L., Gerard R. D., Hammer R. E. and Herz J. (1993). Hypercholesterolemia in low density lipoprotein receptor knockout mice and its reversal by adenovirus-mediated gene delivery. *J. Clin. Invest.* 92, 883-893. 10.1172/JCI116663 8349823 PMC294927

[DMM033852C13] Ishibashi S., Herz J., Maeda N., Goldstein J. L. and Brown M. S. (1994). The two-receptor model of lipoprotein clearance: tests of the hypothesis in “knockout” mice lacking the low density lipoprotein receptor, apolipoprotein E, or both proteins. *Proc. Natl. Acad. Sci. USA* 91, 4431-4435. 10.1073/pnas.91.10.4431 8183926 PMC43799

[DMM033852C14] Isoda K., Sawada S., Ishigami N., Matsuki T., Miyazaki K., Kusuhara M., Iwakura Y. and Ohsuzu F. (2004). Lack of interleukin-1 receptor antagonist modulates plaque composition in apolipoprotein E-deficient mice. *Arterioscler. Thromb. Vasc. Biol.* 24, 1068-1073. 10.1161/01.ATV.0000127025.48140.a3 15059807

[DMM033852C15] Keane T. M., Goodstadt L., Danecek P., White M. A., Wong K., Yalcin B., Heger A., Agam A., Slater G., Goodson M. et al. (2011). Mouse genomic variation and its effect on phenotypes and gene regulation. *Nature* 477, 289-294. 10.1038/nature10413 21921910 PMC3276836

[DMM033852C16] Kennedy A. J., Ellacott K. L. J., King V. L. and Hasty A. H. (2010). Mouse models of the metabolic syndrome. *Dis. Model. Mech.* 3, 156-166. 10.1242/dmm.003467 20212084 PMC2869491

[DMM033852C17] Keren P., George J., Keren G. and Harats D. (2001). Non-obese diabetic (NOD) mice exhibit an increased cellular immune response to glycated-LDL but are resistant to high fat diet induced atherosclerosis. *Atherosclerosis* 157, 285-292. 10.1016/S0021-9150(00)00685-7 11472727

[DMM033852C18] Kleemann R., Zadelaar S. and Kooistra T. (2008). Cytokines and atherosclerosis: a comprehensive review of studies in mice. *Cardiovasc. Res.* 79, 360-376. 10.1093/cvr/cvn120 18487233 PMC2492729

[DMM033852C19] Liang Y., Cucchetti M., Roncagalli R., Yokosuka T., Malzac A., Bertosio E., Imbert J., Nijman I. J., Suchanek M., Saito T. et al. (2013). The lymphoid lineage-specific actin-uncapping protein Rltpr is essential for costimulation via CD28 and the development of regulatory T cells. *Nat. Immunol.* 14, 858-866. 10.1038/ni.2634 23793062

[DMM033852C20] Liang P., Xu Y., Zhang X., Ding C., Huang R., Zhang Z., Lv J., Xie X., Chen Y., Li Y. et al. (2015). CRISPR/Cas9-mediated gene editing in human tripronuclear zygotes. *Protein Cell* 6, 363-372. 10.1007/s13238-015-0153-5 25894090 PMC4417674

[DMM033852C21] Lum Z.-P., Tai I. T., Krestow M., Norton J., Vacek I. and Sun A. M. (1991). Prolonged reversal of diabetic state in NOD mice by xenografts of microencapsulated rat islets. *Diabetes* 40, 1511-1516. 10.2337/diab.40.11.1511 1936609

[DMM033852C41] Lusis A. J. (2000). Atherosclerosis. *Nature* 407, 233-241. 10.1038/35025203 11001066 PMC2826222

[DMM033852C22] Lusis A. J. (2012). Genetics of atherosclerosis. *Trends Genet.* 28, 267-275. 10.1016/j.tig.2012.03.001 22480919 PMC3362664

[DMM033852C23] Mitchell F. (2012). Diabetes: heart is target of autoimmune attack after myocardial infarction in patients with T1DM. *Nat. Rev. Endocrinol.* 8, 504 10.1038/nrendo.2012.117 22751343

[DMM033852C24] Paigen B., Morrow A., Brandon C., Mitchell D. and Holmes P. (1985). Variation in susceptibility to atherosclerosis among inbred strains of mice. *Atherosclerosis* 57, 65-73. 10.1016/0021-9150(85)90138-8 3841001

[DMM033852C25] Pearson J. A., Wong F. S. and Wen L. (2016). The importance of the Non Obese Diabetic (NOD) mouse model in autoimmune diabetes. *J. Autoimmun.* 66, 76-88. 10.1016/j.jaut.2015.08.019 26403950 PMC4765310

[DMM033852C26] Pettersson U. S., Waldén T. B., Carlsson P.-O., Jansson L. and Phillipson M. (2012). Female mice are protected against high-fat diet induced metabolic syndrome and increase the regulatory T cell population in adipose tissue. *PLoS ONE* 7, e46057 10.1371/journal.pone.0046057 23049932 PMC3458106

[DMM033852C27] Plump A. S., Smith J. D., Hayek T., Aalto-Setälä K., Walsh A., Verstuyft J. G., Rubin E. M. and Breslow J. L. (1992). Severe hypercholesterolemia and atherosclerosis in apolipoprotein E-deficient mice created by homologous recombination in ES cells. *Cell* 71, 343-353. 10.1016/0092-8674(92)90362-G 1423598

[DMM033852C28] Ridgway W. M. (2014). A new tool for dissecting genetic control of type 1 diabetes. *Diabetes* 63, 56-58. 10.2337/db13-1370 24357698

[DMM033852C29] Ross R. (1999). Atherosclerosis is an inflammatory disease. *Am. Heart J.* 138, S419-S420. 10.1016/S0002-8703(99)70266-8 10539839

[DMM033852C30] Shao Y., Guan Y., Wang L., Qiu Z., Liu M., Chen Y., Wu L., Li Y., Ma X., Liu M. et al. (2014). CRISPR/Cas-mediated genome editing in the rat via direct injection of one-cell embryos. *Nat. Protoc.* 9, 2493-2512. 10.1038/nprot.2014.171 25255092

[DMM033852C31] Smedbakken L. M., Halvorsen B., Daissormont I., Ranheim T., Michelsen A. E., Skjelland M., Sagen E. L., Folkersen L., Krohg-Sorensen K., Russell D. et al. (2012). Increased levels of the homeostatic chemokine CXCL13 in human atherosclerosis-Potential role in plaque stabilization. *Atherosclerosis* 224, 266-273. 10.1016/j.atherosclerosis.2012.06.071 22840692

[DMM033852C32] Smith J. (2003). Quantitative trait locus mapping for atherosclerosis susceptibility. *Curr. Opin. Lipidol.* 14, 499-504. 10.1097/00041433-200310000-00011 14501589

[DMM033852C33] Srivastava U., Paigen B. J. and Korstanje R. (2012). Differences in health status affect susceptibility and mapping of genetic loci for atherosclerosis (fatty streak) in inbred mice. *Arterioscler. Thromb. Vasc. Biol.* 32, 2380-2386. 10.1161/ATVBAHA.112.255703 22837474 PMC3563286

[DMM033852C34] Tabibiazar R., Wagner R. A., Spin J. M., Ashley E. A., Narasimhan B., Rubin E. M., Efron B., Tsao P. S., Tibshirani R. and Quertermous T. (2005). Mouse strain-specific differences in vascular wall gene expression and their relationship to vascular disease. *Arterioscler. Thromb. Vasc. Biol.* 25, 302-308. 10.1161/01.ATV.0000151372.86863.a5 15550693

[DMM033852C35] Vanden Berghe T., Hulpiau P., Martens L., Vandenbroucke R. E., Van Wonterghem E., Perry S. W., Bruggeman I., Divert T., Choi S. M., Vuylsteke M. et al. (2015). Passenger mutations confound interpretation of all genetically modified congenic mice. *Immunity* 43, 200-209. 10.1016/j.immuni.2015.06.011 26163370 PMC4800811

[DMM033852C36] Velasco E., Infante M., Durán M., Pérez-Cabornero L., Sanz D. J., Esteban-Cardeñosa E. and Miner C. (2007). Heteroduplex analysis by capillary array electrophoresis for rapid mutation detection in large multiexon genes. *Nat. Protoc.* 2, 237-246. 10.1038/nprot.2006.482 17401359

[DMM033852C37] Warnatsch A., Ioannou M., Wang Q. and Papayannopoulos V. (2015). Inflammation. Neutrophil extracellular traps license macrophages for cytokine production in atherosclerosis. *Science* 349, 316-320. 10.1126/science.aaa8064 26185250 PMC4854322

[DMM033852C38] Yang H., Wang H. and Jaenisch R. (2014). Generating genetically modified mice using CRISPR/Cas-mediated genome engineering. *Nat. Protoc.* 9, 1956-1968. 10.1038/nprot.2014.134 25058643

[DMM033852C39] Zernecke A. and Weber C. (2010). Chemokines in the vascular inflammatory response of atherosclerosis. *Cardiovasc. Res.* 86, 192-201. 10.1093/cvr/cvp391 20007309

