# Supplementary material for: A severe atherosclerosis mouse model on the resistant NOD background
Source: Dis Model Mech. 2018 Oct 8;11(10):dmm033852. doi: 10.1242/dmm.033852 (PMC6215432; doi:10.1242/dmm.033852)
Supplement: Supplementary information [file dmm-11-033852-s1.pdf]

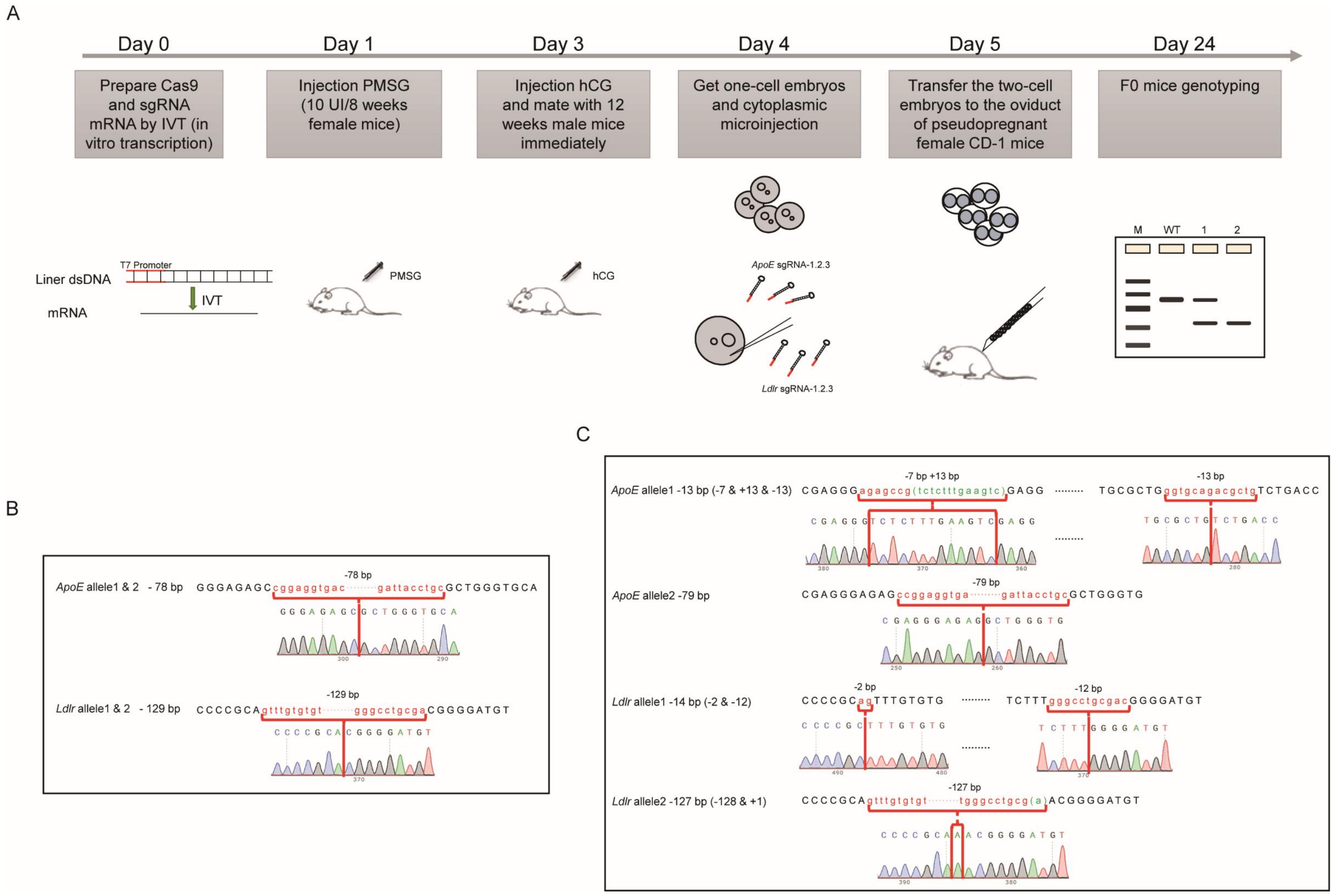

Fig. S1. Genetic ablation of ApoE and LDLR in NOD mouse by CRISPR/Cas9 system.
(A) Workflow for generation of *ApoE* and *LDLR* knockout mice using the CRISPR/Cas9 system. Experimental steps involved sgRNA and Cas9 mRNA synthesis, injection of PMSG and hCG hormones for superovulation, collection of embryos, microinjection and founder identification by fluorescent PCR.
(B)(C) Sanger sequencing result of mutant-1 (KO#1) and mutant-2 (KO#2) in F0 animals. The deletions were annotated in red letters and insertions in green letters by alignment with wildtype sequences involving *ApoE* and *LDLR* genes.

Disease Models & Mechanisms • Supplementary information

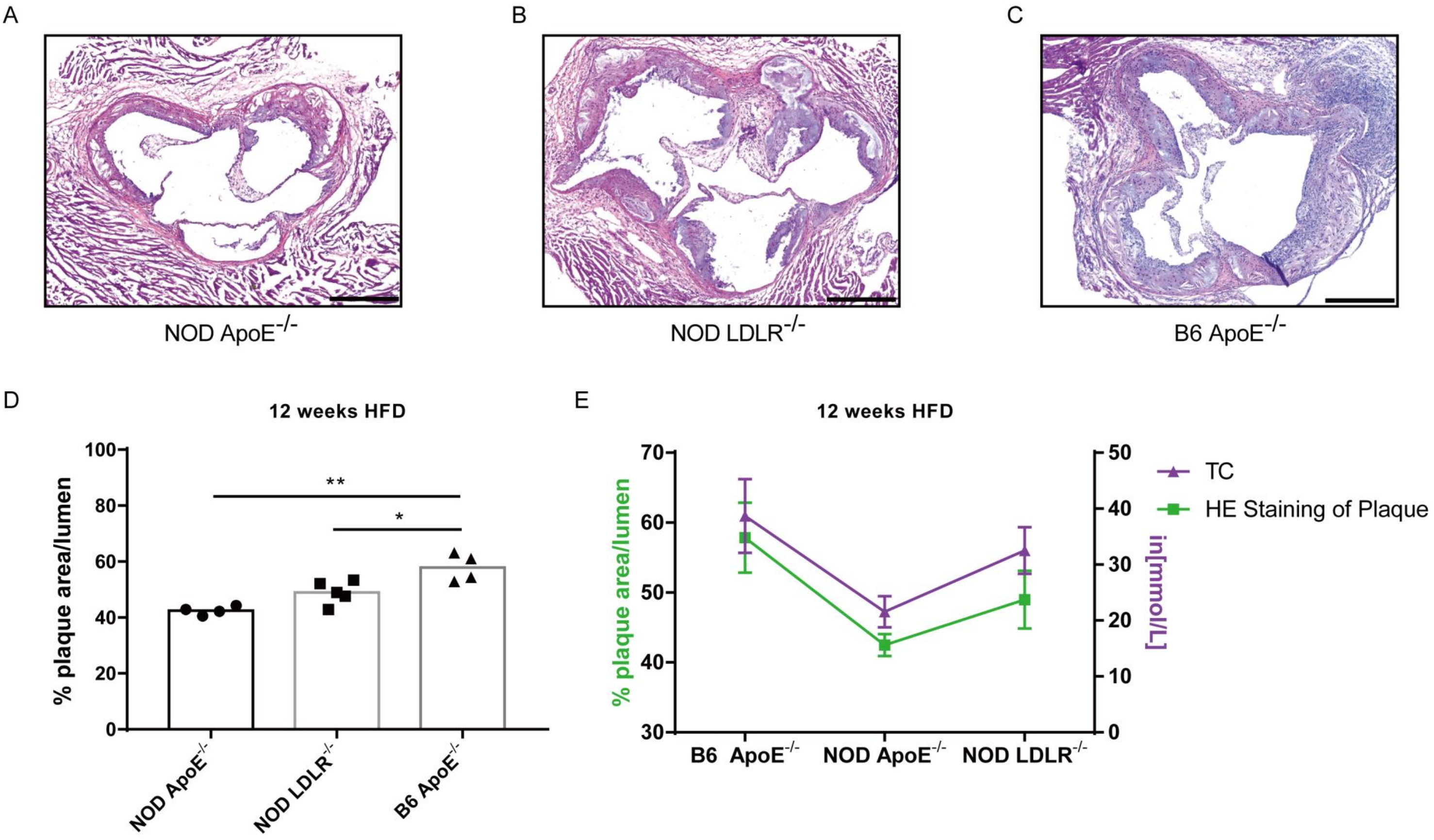

Fig. S2. Atherosclerosis development in ApoE or LDLR deficient NOD mice.

(A-C) The representative sections of aortic sinus from three types of mice, scale bar=400μm.

(D) Quantitation of plaque area relative to the area of the aortic sinus region after staining with H&E , NOD ApoE$^{-/-}$(n=4, male=2, female=2), NOD LDLR$^{-/-}$(n=5 , male=3, female=2) and B6 ApoE$^{-/-}$ (n=4 , male=2, female=2) that were fed with HFD for 12 weeks.

(E) The correlation between atherosclerosis burden and that of their corresponding total cholesterol (TC).

Data were collected from two independent experiments and statistical analyses were performed by two-tailed, unpaired Student's t-test: * p<0.05, ** p<0.01, *** p<0.001.

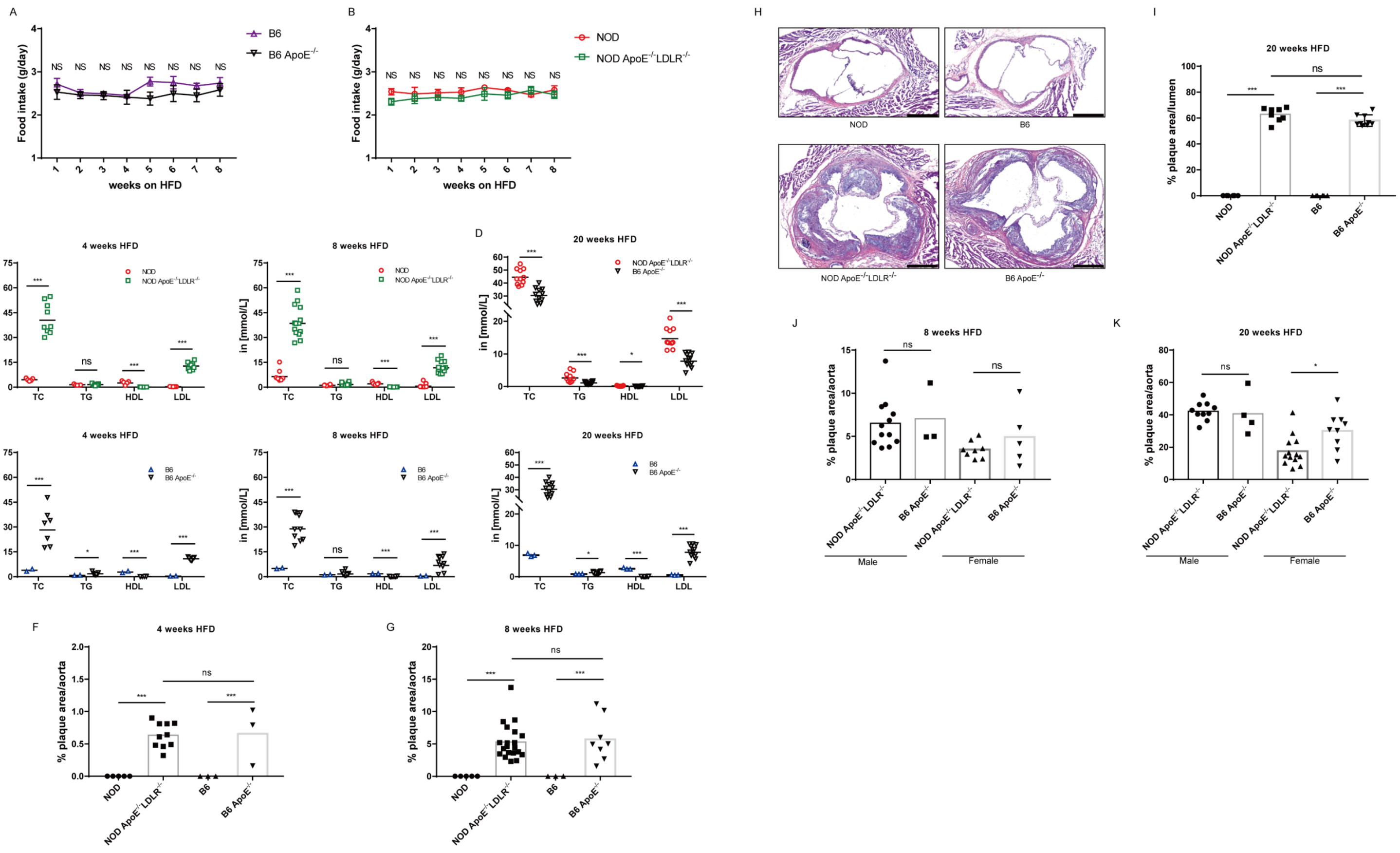

Fig. S3. Hyperlipidemia and atherosclerosis in the double knockout NOD ApoE$^{-/-}$LDLR$^{-/-}$mice.
(A-B) Food intake of B6, B6 ApoE$^{-/-}$andNOD, NOD ApoE$^{-/-}$LDLR$^{-/-}$animals fed with HFD for 8 weeks (n=7-16).
(C) Blood TC, TG, HDL and LDL concentrations of NOD ApoE$^{-/-}$LDLR$^{-/-}$animals fed with HFD for 4, 8 weeks with NOD wild type controls (n=4-14).
(D) Comparisons of blood TC, TG, HDL and LDL concentrations between NOD ApoE$^{-/-}$LDLR$^{-/-}$and B6 ApoE$^{-/-}$animals on HFD for 20 weeks (n=9-12).
(E) Comparisons of blood TC, TG, HDL and LDL concentrations between B6 animals and B6 ApoE$^{-/-}$mice fed with HFD for 4, 8, 20 weeks (n=2-12).
(F-G) Quantitative analysis of en face lesion area of NOD ApoE$^{-/-}$LDLR$^{-/-}$and B6 ApoE$^{-/-}$animals fed with HFD for 4 or 8 weeks with B6 and NOD wild type controls (n=3-20). (The number of animals used in the 4 HFD experiment were: NOD, n=5 (male=3, female=2); NOD ApoE$^{-/-}$LDLR$^{-/-}$, n=10 (male=6, female=4); B6, n=3 (male=2, female=1); B6 ApoE$^{-/-}$, n=3 (male=2, female=1). The number of animals used in the 8 HFD experiment were: NOD, n=5 (male=3, female=2); NOD ApoE$^{-/-}$LDLR$^{-/-}$, n=20 (male=12, female=8); B6, n=3 (male=1, female=2); B6 ApoE$^{-/-}$, n=8 (male=3, female=5).)
(H) Representative images of H&E stained sections of the aortic sinus from NOD ApoE$^{-/-}$LDLR$^{-/-}$ and B6 ApoE$^{-/-}$animals fed with HFD for 20 weeks with B6 and NOD wild type controls , scale bar=400μm.
(I) Quantitation of plaque areas sections of aortic sinus from miceused in (H) (The number of animals used in the experiment were: NOD, n=6 (male=3, female=3); NOD ApoE$^{-/-}$LDLR$^{-/-}$, n=8 (male=5, female=3); B6, n=6 (male=3, female=3); B6 ApoE$^{-/-}$, n=9 (male=4, female=5).).
(J-K) Comparisons of en face lesion area of NOD ApoE$^{-/-}$LDLR$^{-/-}$and B6 ApoE$^{-/-}$animals fed with HFD for 8 or 20 weeks (using the same mice from fig. S3G and fig. 3E).
The data was collected from two indenpendent experiments. Statistics by two-tailed, unpaired Student's t-test: *p <0.05, ** p<0.01, ***p<0.001.

Disease Models & Mechanisms • Supplementary information

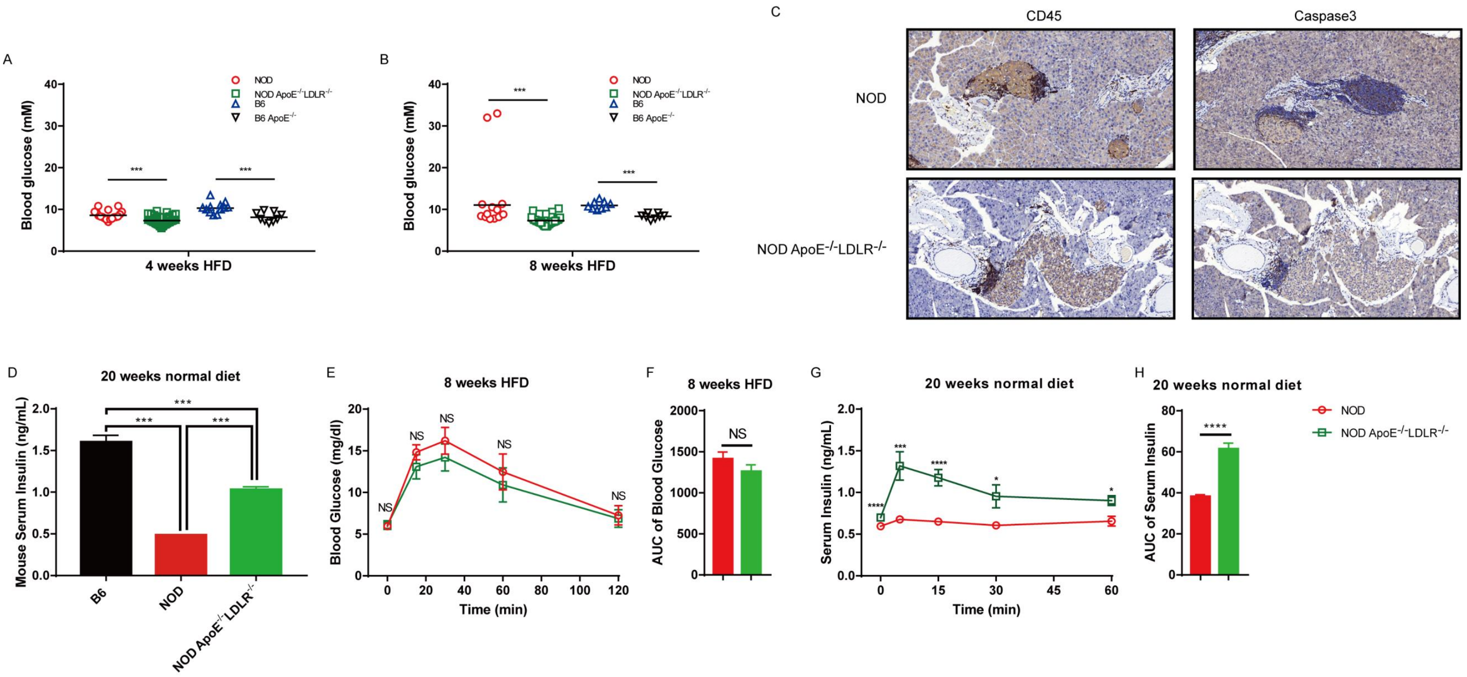

Fig. S4. Blood glucose, serum insulin, GTT and GSIS in NOD and NOD ApoE$^{-/-}$LDLR$^{-/-}$ mice on normal and high fat diet.

(A) Blood glucose levels (mM) levels in NOD ApoE$^{-/-}$LDLR$^{-/-}$ and B6 ApoE$^{-/-}$ animals after 4 weeks of HFD, with controls of NOD and B6 mice (n=9-54).

(B) Blood glucose levels (mM) levels in NOD ApoE$^{-/-}$LDLR$^{-/-}$ and B6 ApoE$^{-/-}$ animals after 8 weeks of HFD, with controls of NOD and B6 mice (n=8-42).

(C) Representative immunostained pancreas from NOD and NOD ApoE$^{-/-}$LDLR$^{-/-}$mice fed with normal diet using specific antibodies against CD45 and Caspase3.

(D) Serum insulin levels of NOD, NOD ApoE$^{-/-}$LDLR$^{-/-}$and B6 after 20 weeks of normal diet (n=5-6).

(E) Glucose tolerance tests (2 g/kg total body weight) was performed in overnight-fasted mice. Blood glucose levels of NOD, NOD ApoE$^{-/-}$LDLR$^{-/-}$mice were measured at the indicated time-points after 8 weeks of HFD (n=8-10).

(F) The areas under the curves (AUC) of blood glucose (data from E).

(G) GSIS analysis in vivo in 5-month-old NOD and NOD ApoE$^{-/-}$LDLR$^{-/-}$ mice (n=6).

(H) The areas under the curves (AUC) of serum insulin (data from G).

Statistics by two-tailed, unpaired Student's t-test: * $p<0.05$, ** $p<0.01$, *** $p<0.001$.

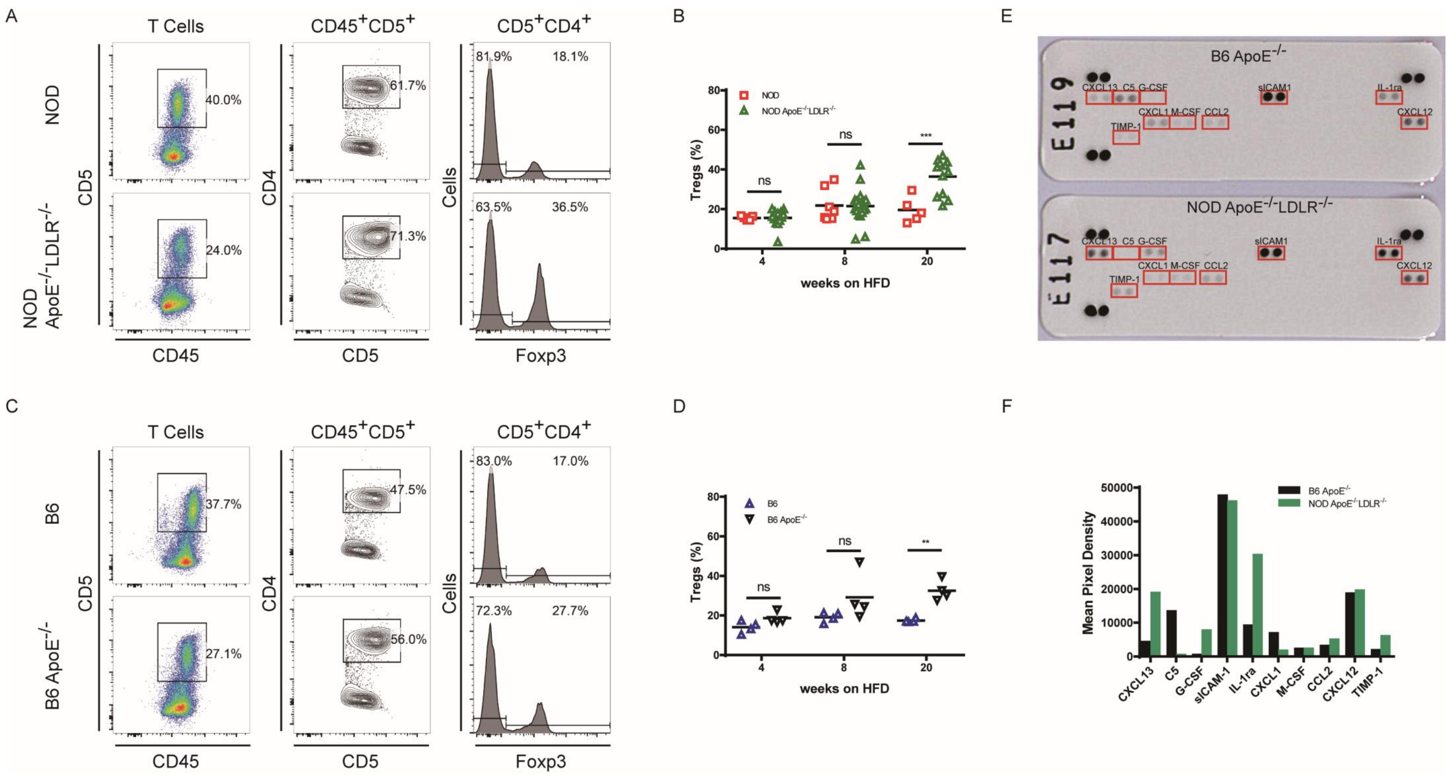

Fig. S5. Immune response of in NOD ApoE$^{-/-}$LDLR$^{-/-}$ mice to hyperlipidemia.

(A) The gating method for regulatory T cells in the splenocytes of NOD ApoE$^{-/-}$LDLR$^{-/-}$ with NOD as control.

(B) Frequency of Foxp3 positive cells in CD4 T cells of NOD and NOD ApoE$^{-/-}$LDLR$^{-/-}$ mice on 20 weeks of HFD (n=5-20).

(C) The gating method for regulatory T cells in the splenocytes of B6 ApoE$^{-/-}$ and B6 control.

(D) Frequency of Foxp3 positive cells in CD4 T cells of B6 ApoE$^{-/-}$ and B6 mice on HFD for 20 weeks (n=4).

(E) Serum cytokines in B6 ApoE$^{-/-}$ and NOD ApoE$^{-/-}$LDLR$^{-/-}$ fed on HFD for 20 weeks. 100 µL serum samples collected from three mice from each group and analyzed by the Proteome Profiler Mouse Cytokine Array Kit. Data shown are from a five minute exposure.

(F) Quantification of the cytokine expression in samples used in (E).

Statistics by two-tailed, unpaired Student's t-test: * $p<0.05$, ** $p<0.01$, *** $p<0.001$.

Disease Models & Mechanisms • Supplementary information

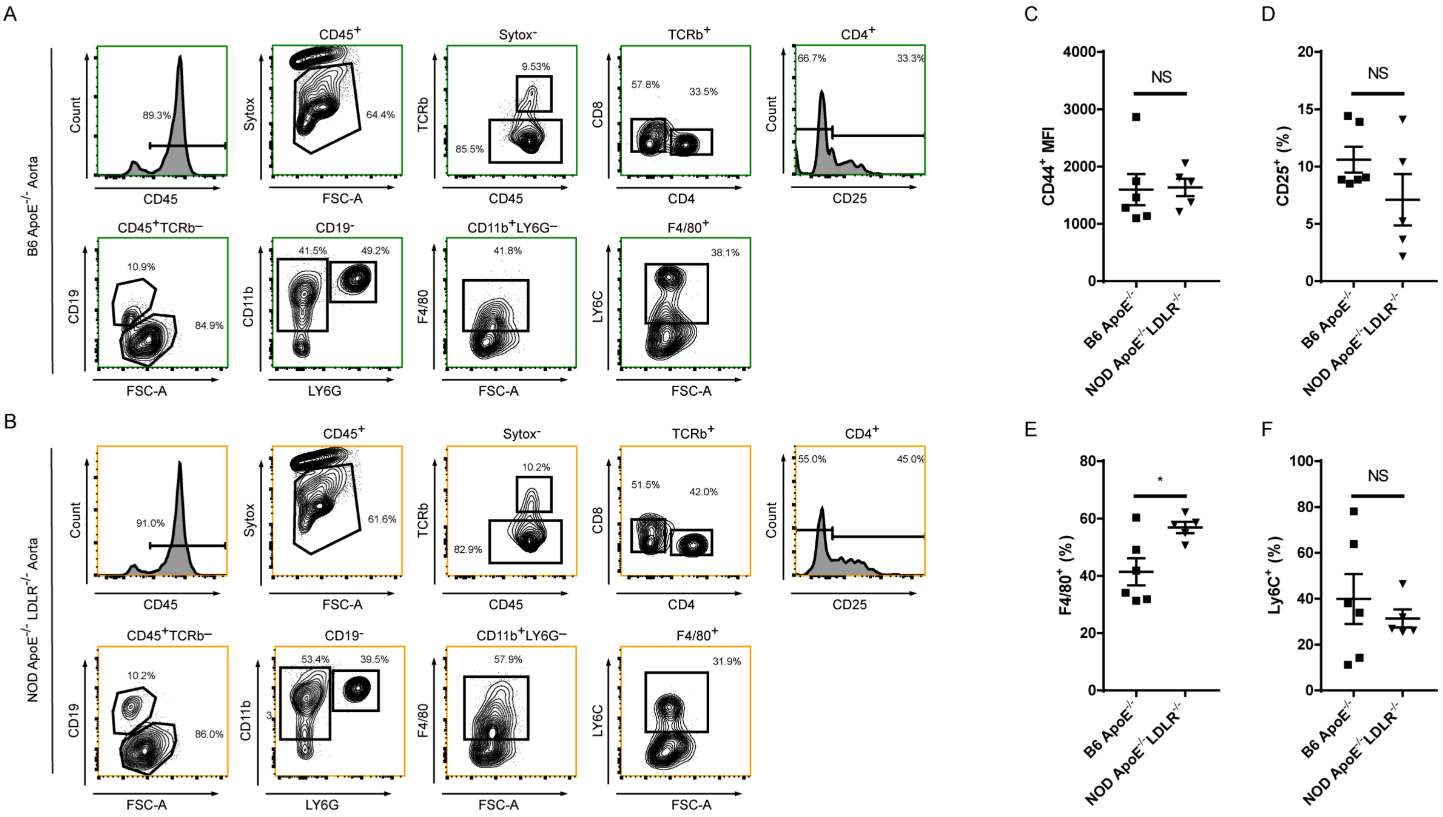

Fig. S6. Immunophenotyping of aorta in NOD ApoE$^{-/-}$LDLR$^{-/-}$ mice.

(A-B) The gating method for immune cells in the aorta of B6 ApoE$^{-/-}$ and NOD ApoE$^{-/-}$LDLR$^{-/-}$ mice on 8 weeks of HFD.

(C) MFI of CD44 positive cells in B6 ApoE$^{-/-}$ and NOD ApoE$^{-/-}$LDLR$^{-/-}$ mice on 8 weeks of HFD.

(D) Frequency of CD25 positive cells in CD4 T cells of B6 ApoE$^{-/-}$ and NOD ApoE$^{-/-}$LDLR$^{-/-}$ mice on 8 weeks of HFD.

(E) Frequency of F4/80 positive cells in CD11b^{+}LY6G^{-} cells of B6 ApoE$^{-/-}$ and NOD ApoE$^{-/-}$LDLR$^{-/-}$ mice on 8 weeks of HFD.

(F) Frequency of LY6C positive cells in F4/80^{+} cells of B6 ApoE$^{-/-}$ and NOD ApoE$^{-/-}$LDLR$^{-/-}$ mice on 8 weeks of HFD.

The analyses above were performed with the same animals (n=5-6). Statistics by two-tailed, unpaired Student's t-test: * $p<0.05$, ** $p<0.01$, *** $p<0.001$.

Disease Models & Mechanisms • Supplementary information

Table S1. Primers for genotyping

primer	Sequence	labeled	Amplicon length
LDLR-F PCR-F	AGACGTGCTCCCAGGATG	FAM	230 bp
LDLR-F PCR-R	GTTCTGTGGCCACTCATCG		
LDLR-S PCR-F	CCACCCATGGCTCTGCTAAA		593 bp
LDLR-S PCR-R	CCTTTCTGGGCAGTCTGGTT		
ApoE-F PCR-F	CGAGGGTGAAAGAGCTGGAC	HEX	268 bp
ApoE-F PCR-R	GCCTCCAGACCCACTTCAAA		
ApoE-S PCR-F	AAGGCAGGAGGATTCAAGGC		447 bp
ApoE-S PCR-R	GCCTAGTCTCGGCTCTGAAC		

Table S2 A. Antibody staining to analyze regulatory T cells

Name	Fluorochrome	Company
Anti mouse-CD45	APC-eFlour780	eBioscience
Anti mouse-CD4	Biotin	BD Pharmingen
Streptavidin	BV605	Biolegend
Anti mouse-CD5	eFluor450	eBioscience
Anti mouse-FOXP3	PE	eBioscience

Table S2 B. Antibody staining to analyze phenotype of moncytes

Name	Fluorochrome	Company
Anti mouse-CD45	APC-eFlour780	eBioscience
Anti mouse-CD5	PE-Cyanine7	eBioscience
Anti mouse-CD19	PE-Cy5.5	eBioscience
Anti mouse-CD11b	APC	eBioscience
Anti mouse-CD11c	PE	eBioscience
Anti mouse-Ly6G	Alexa Fluor700	BD Pharmingen
Anti mouse-Ly6C	eFluor450	eBioscience

Table S2 C. Antibody staining to analyze phenotype of aorta

Name	Fluorochrome	Company
Anti mouse-CD45	APC-eFlour780	eBioscience
Anti mouse-TCRb	FITC	eBioscience
Anti mouse-CD44	BV605	eBioscience
Anti mouse-CD19	PE-Cy5.5	eBioscience
Anti mouse-CD11b	APC	eBioscience
Anti mouse-CD4	eFluor450	eBioscience
Anti mouse-CD8	Alexa 700	eBioscience
Anti mouse-Ly6G	Alexa Fluor700	BD Pharmingen
Anti mouse-Ly6C	eFluor450	eBioscience
Anti mouse-F4/80	PE-Cyanine7	eBioscience
Sytox	V500	eBioscience

Disease Models & Mechanisms • Supplementary information
